# Pre-Treatment with Estradiol, But not Progesterone, Exacerbates DSS Colitis: Dysregulated Innate Immunity and Impaired Epithelial Damage Response

**DOI:** 10.1007/s10753-026-02554-y

**Published:** 2026-07-02

**Authors:** Anja Hjelt, Lauri Polari, Heli Jokela, Pia Rantakari, Heidi Gerke, Diana M. Toivola, Claes Ohlsson, Matti Poutanen, Jorma Määttä

**Affiliations:** 1 Institute of Biomedicine, University of Turku, Kiinamyllynkatu 10, Turku, 20520 Finland; 2 Turku Bioscience Center, University of Turku and Åbo Akademi University, Tykistökatu 6, Turku, 20520 Finland; 3 Department of Natural and Health Sciences, Faculty of Science and Engineering, Biochemistry and Cell Biology, Åbo Akademi University, Turku, Finland; 4 InFLAMES Research Flagship Center, BioCity, Tykistökatu 6A, Turku, 20520 Finland; 5 Department of Internal Medicine and Clinical Nutrition, The Sahlgrenska Osteoporosis Centre, Institute of Medicine, Sahlgrenska Academy, University of Gothenburg, Gothenburg, Sweden; 6 Turku Center for Disease Modeling, University of Turku, Turku, Finland

**Keywords:** Estradiol, Estrogen receptor, Inflammation, DSS colitis, Innate immunity

## Abstract

Epidemiological data have indicated an increased risk of inflammatory bowel disease associated with the female sex and contraceptive hormones. However, in preclinical models of estradiol in colitis, results have been inconsistent, and both alleviating and aggravating effects have been reported. Previously, we suggested that the estrogen receptor α mediates inflammation in the colon. Here, we investigated the effects of estradiol and progesterone in dextran sulphate sodium-induced colitis, using sham-operated or ovariectomized female mice with or without hormonal supplementation initiated three weeks before inflammation. We found that sham-operated, or ovariectomized mice supplemented either with only estradiol or estradiol and progesterone exhibited more severe intestinal inflammation compared to ovariectomized mice at day 7 of colitis. Simultaneously, progesterone supplementation of ovariectomized mice did not affect the inflammatory status. Estrogen receptors were expressed in several different cell types of the colonic mucosa, although at low levels. The estrogen receptor α was observed in the epithelium as well as in mononuclear phagocytes and T and B cells. The estrogen receptor β was found in the epithelium, and the G protein-coupled estrogen receptor in the *lamina propria* and vessel structures. Further, hormone depletion increased homeostatic immune cells, while estradiol exposure altered the expression of 791 genes involved in pathways promoting the antimicrobial response, immune cell activation, and metabolism related to epithelial integrity. With our results, we are the first to present strong and detailed evidence of estradiol as an inflammatory agent in the colon of female mice when allowing the appearance of longer-term effects.

**Supplementary Information:**

The online version contains supplementary material available at https://doi.org/10.1007/s10753-026-02554-y.

## Introduction

The pathophysiology of inflammatory bowel disease (IBD) is complex, involving multiple mucosal cell types and processes, with both genetic and environmental factors triggering dysregulation. The two main forms of IBD, Crohn´s disease (CD) and ulcerative colitis (UC), manifest differently as lesions in the gastrointestinal system, but share pathophysiology such as dysbiosis, impairment of the intestinal barrier, and disproportionate activation of both the adaptive and the innate immune system [1]. The use of estradiol (E2), as well as progestogen-containing contraceptives, has been linked to a treatment duration-dependent risk increase of both UC and CD [2, 3]. Additionally, female sex is associated with an increased disease risk [4]; the onset of CD is more common in females, beginning from the mid-twenties [5].

Expression of the nuclear estrogen receptors α and β (ERα, encoded by *Esr1*, and ERβ by *Esr2*), as well as the transmembrane G protein-coupled estrogen receptor (GPER, encoded by *Gper1*), has been reported in the human and murine colon, with expression levels altering in intestinal inflammation [6–9]. However, reports of inconsistent data from studies measuring the binding of estrogenic ligands to transfected constructs of GPER led to some uncertainty on whether GPER is an estrogen receptor [10–13]. Nonetheless, studies utilizing genetic depletion of GPER in mice indicate an involvement of GPER in the signalling of E2 or synthetic agonist G1 in vivo [14–16]. The classic, genomic estrogen response is initiated after ligand binding, receptor dimerization, and nuclear translocation. In the nucleus, receptor dimers assemble transcription factor complexes that bind to available estrogen response elements (ERE) in the genome. Ovarian hormones can produce context-dependent pro- and anti-inflammatory responses, which has been reflected in inflammation studies in both humans and mice [17].

The dextran sulphate sodium (DSS)–induced colitis model is a commonly used and non-invasive method to investigate inflammation following barrier breakdown. DSS affects especially the distal colon but also alters barrier function in the proximal colon and the ileum [18, 19]. The DSS colitis model recapitulates the features of ER signalling in human IBD: treatment duration-dependent worsening of intestinal inflammation in young adult females [2, 3, 20, 21] and a protective effect of ERβ, which commonly is associated with maintained barrier function and downregulated in intestinal inflammation [8, 9, 20, 22–24]. In another chemically induced colitis model, 2,4,6-trinitrobenzenesulfonic acid (TNBS) colitis, the protective effect of ERβ was not observed [25].

The divergent effects of E2 in DSS colitis are apparent in the literature. For instance, when the DSS model was used in a colitis-associated cancer study, E2 pre-treatment for 1 week before induction aggravated inflammation in male or ovariectomized (OVX) female mice [21]. Additionally, in our previous work, we demonstrated that endogenous ovarian hormones, as well as three weeks of E2 pre-treatment, augmented the inflammatory effects of the 7-day DSS challenge, worsening colitis in female FVB/n mice [20]. Furthermore, ovariectomy or prophylactic treatment with an experimental ERα modulator reduced inflammation [19, 20], further suggesting that E2 signalling through ERα exacerbates colitis. However, E2 pre-treatment studies have not been as commonly performed in DSS models as studies where E2 has been administered for 5–12 days during or after DSS colitis induction. In contrast to the pre-treatment studies, later E2 treatment was associated with attenuated colitis most often in male mice, but also in females [6, 26–30]. Thus, the timing of estrogen exposure in relation to the onset of inflammation might be a factor influencing the outcome.

In addition to the receptor subtype-specific and temporal effects, sex differences may influence the effects of estrogen in colitis. Males are generally more severely affected by colitis, and sex-specific effects of ER signalling have been reported. An inflammatory effect of ERβ depletion, together with an anti-inflammatory effect of ERα depletion, were reported in female mice, while the opposite was observed in males [24]. Nevertheless, ERα signalling can also be detrimental in male mice, and treatment with a specific ERα agonist increased inflammation, even though the treatment was initiated after the onset of inflammation [7]. Last, ER signalling in different cell types might influence the response, although hitherto published results of cell type-specific ER depletion have aligned with those seen in mice with global ER depletion. When either T cell-specific ERα depletion [31], or intestinal epithelium-specific ERα [32] and ERβ depletion [9] were performed in mice, ERβ signalling was observed to be protective, while ERα signalling aggravated inflammation in female colitis. This suggests a broad estrogen effect in several cell types, as modulation of both epithelial and immune cell estrogen receptors influenced colitis outcomes.

Similar to E2, previous literature also presents diverse effects of progesterone (P4) in inflammation. For instance, progestogens used in contraceptives are associated with a modest risk increase for ulcerative colitis [3]. Also, the anti-inflammatory experimental ERα antagonist used in our previous studies was indicated to also bind and inhibit the progesterone receptor (PGR), although with lower affinity [20, 33]. Nonetheless, permeability-decreasing and anti-inflammatory effects have also been associated with P4 [34]. Intestinal permeability, which is regulated by epithelial tight junctions and increases in inflammation, was shown to inversely correlate with serum P4 concentration in early human pregnancy, and colonocyte tight junction protein was upregulated by P4 treatment in human ex vivo tissues [35]. Further, P4 decreased TNBS-induced colitis in male rats [36]. In the only previous study available where activation of both ER and PGR was investigated in the colon, E2 increased tumours induced by azoxymethane and DSS in female mice. In the same study, progestogen alone or in combination with E2 did not influence the susceptibility of developing colitis-associated cancer [21]. However, no previous studies have examined the effects of ER and PGR activation in a disease model of IBD. Supplementing OVX mice with a combination of E2 and P4, rather than E2 alone, better reflects both the physiological hormonal environment and the use of combination contraceptives.

Here, we investigated the effects of ovariectomy alone or in combination with E2, P4, or E2 + P4 supplementation initiated 3 weeks before the induction of colitis using DSS. With this, we wish to expand the knowledge on E2 and P4 pre-treatment in female acute intestinal inflammation, as only two studies have previously reported increased colitis or colitis-associated cancer after E2 pre-treatment, while shorter experimental setups, where E2 was administered simultaneously or after DSS, yielded opposite results [20, 21, 27, 28]. Additionally, as effects of both global and cell-type-specific depletion of ERs were previously indicated in colitis [9, 24, 31, 32] we aimed to explore the estrogen-responsive cell types in inflammation and establish links to the transcriptional mechanisms underlying the hormonal effect in female colitis.

## Methods

### Animal Experiment

Two experiments were carried out with identical treatments, but sampling and analyses varied between experiments (total *n* = 34 in experiment 1, *n* = 30 in experiment 2). Janvier Labs FVB/NHanHsd female mice were purchased from Envigo at 6 to 7 weeks of age. Mice were ovariectomized or sham-operated after one week of acclimatizing. Anesthesia was induced using isoflurane (induction 4–5%, maintenance 1.7–2.0%), and for analgesia, buprenorphine 0.05 mg/kg and karprofen 5 mg/kg were administered *s.c.* once/day for 3 days. Some of the OVX mice were supplemented with E2 and P4. Hormone-containing or empty control implants (MedRod^®^, PreclinApps Ltd.) were inserted *s.c.* simultaneously with the ovariectomy, after which 2.5 weeks (experiment 1) or 3.5 weeks (experiment 2) were reserved for recovery. Mice were allocated to treatment groups by a technician outside the research group, and they were housed in cages with one mouse from each treatment group. Groups: sham-operated (experiment 1: *n* = 2, experiment 2: *n* = 6), OVX, OVX+E2 0.25 µg/d [approximately 10 µg/kg/day], OVX+P4 15 µg/day [approximately 600 µg/kg/day], or OVX+E2 0.25 µg/d + P4 15 µg/day (experiment 1: *n* = 8, experiment 2: *n* = 6 in each group). The number of mice used in each analysis is indicated in the figure legends. To induce colitis, all mice were administered DSS (40 kDa, TdB Labs), dosing 2.5% w/v in drinking water for 7 days. Weight and disease activity were monitored before and during the experiment. Mice were sacrificed at 10–11 weeks of age by CO_2_ on day 7 of DSS treatment. Colon length was measured from the distal cecum to the rectum, after which the middle to distal parts were collected. The whole uterus without ovaries was weighed after sacrifice to verify the E2 effect. Specific pathogen-free housing was provided by the Central Animal Laboratory at the University of Turku. Tunnels, huts, and nesting material were provided as environmental enrichment. The mice were fed a soy-free diet (SDS RM3 Soy-free, Teklad). A priori exclusion criteria were animal welfare issues leading to the death of the animal before DSS day 7. However, no mice were euthanized before the end of the experiment.

### Disease and Total Colitis Score

Symptoms of colitis were scored from 0 to 4 (with a maximum of 12) based on the amount of weight loss, fecal consistency, and the appearance of blood in the feces. For total inflammation scores, the symptom score was added to a histological inflammation score calculated from all histological samples of the first experiment and partially from the second experiment, for which middle and distal colon sections were visually analysed from digital scans including the whole sample and scored from 0 to 3 (max 15) based on the amount of immune infiltration, edema, epithelial erosion, and crypt hyperproliferation, as previously described [20]. The analysis was performed by a researcher unaware of the treatment of each sample.

### Serum Collection and Hormone Level analysis

At sacrifice, blood was collected through heart puncture, allowed to coagulate, and centrifuged at 1500 g for 10 min to collect serum. Serum samples were stored at −80 °C until hormone levels were measured by gas chromatography-tandem mass spectrometry, as previously described [37].

### Immune Cell Isolation

Longitudinal middle and distal colon tissue samples from the second experiment were cleared from feces in ice-cold PBS and then collected in Hank´s buffer saline solution (HBSS) containing 2% inactivated fetal bovine serum (iFBS). Tissue was incubated in HBSS containing 2 mM EDTA (Sigma) at 37 °C on an orbital shaker (240 rpm) for 15 min, and again in fresh HBSS/EDTA for 30 min, after which tubes were shaken vigorously. For digestion, 1 mg/ml collagenase VIII from *Clostridium histolyticum* (Merck) and 10 µg/ml DNase I (Roche/Merck) were added to RPMI 1640 medium containing 10% iFBS, 15 mM HEPES, and the samples were incubated on an orbital shaker (240 rpm) at 45 min 37 °C. All reagents were acquired from Gibco, Thermo Fisher, unless otherwise mentioned. Cells were suspended in FACS buffer (PBS containing 2% iFBS, 1 mM EDTA, and 1% NaN) and the suspension was filtered through a 70 μm cell strainer (Corning^®^).

### Flow Cytometry Analyses

Dead cells were dyed using eBioscience™ Fixable Viability Dye eFluor™ 780 (Invitrogen), diluted 1:1000 in PBS for 30 min. Unspecific binding was blocked by Mouse BD Fc Block™ (Purified Rat Anti-Mouse CD16/CD32, Clone 2.4G2, BD Biosciences) diluted 1:100 in FACS buffer for 10 min before adding antibody suspension.

Two panels of antibodies were stained and measured in parallel to investigate changes in immune populations. The first panel included leukocyte marker CD45, neutrophil marker Ly6G, monocyte marker Ly6C, and monocyte-macrophage markers F4/80, CD206, MHCII, CX3CR1, CD11b, and dendritic cell marker CD11c.

The second panel included CD45, T cell markers CD4, CD8, γδTCR, and B cell marker CD19. The cells were fixed using Fixation Buffer (BD Biosciences) and stored at 4 °C until cell sorting and analysis. Flow cytometry was performed using LSR Fortessa (405 nm, 488 nm, 561 nm, 640 nm lasers) and analyzed with FlowJo™ v10.8, both from BD Biosciences. Antibodies, dilutions, and fluorochromes, as well as gating strategies, are provided in the supplementary information (Supplemental Material 2 and Supplemental Fig. 2 A). Three samples were excluded from the analysis because of sample quality issues (one sham, two OVX).

### RT-qPCR

Tissues were digested as described above, and separate samples were taken before the filtration step and lysed. RNA was extracted using the Nucleospin RNA and protein kit (Macherey-Nagel). RNA quantity and quality were measured using a Nano-Drop Microvolume Spectrophotometer (Thermo Fisher), with A260/A230 readings above 2. Reverse transcription was performed using the High-Capacity cDNA Reverse Transcription Kit and the VeritiPro Thermal Cycler. 20 ng cDNA/reaction, TaqMan gene expression assays (Supplemental Material 2) and TaqMan™ Universal Master Mix II, no UNG or TaqMan Fast Advanced master mix, no UNG (all from Applied Biosystems, ThermoFisher) were used for RT-qPCR in a CFX Opus 96 Real-Time PCR System, operated by the CFX Maestro™ software (all from BIO-RAD). *Rn18s* was used as the reference gene, and data were analysed using the ΔΔCq method.

### RNA Sequencing, Differential Gene Expression Analysis, and Gene Ontology

Longitudinal middle and distal colon whole-tissue samples from the first experiment were cleaned from feces in ice-cold PBS, then snap-frozen in liquid N_2_ and homogenized (UltraTurrax, IKA). Otherwise, the total RNA from the distal colon was extracted as described above. Before sequencing, sample quality was ensured using the Analytical Fragment Analyzer (Agilent), and all samples measured RQN values between 7.5 and 10, with an average fragment size of 353 bp, and no fragmentation below 150 bp. Sample concentration was measured with Qubit^®^/Quant-IT^®^ Fluorometric Quantitation (Life Technologies). The library was prepared using the Stranded mRNA Preparation and Ligation Kit (Illumina). Sequencing was performed using the Illumina NovaSeq 6000 instrument for paired-end sequencing (2 × 50 bp) and a read depth of 11.6–16.4 M with 2 lanes/sample.

Sequencing read quality was assessed using FastQC (v.0.11.8) and MultiQC (v.1.10). Sequencing data were analyzed using an in-house pipeline implemented in R v.4.1.0 [38] and Bioconductor v.3.13 [39]. Reads were aligned to the mm10 reference genome using Rsubread (v.2.6.4, gene annotation) and align packages. Read assignment to genomic features was performed using featureCount in Rsubread (v.2.6.4). Gene counts were normalized using edgeR (v.3.34.1). Counts per million (CPM) was used for comparing differential gene expression between samples, and transcripts per million (TPM), according to the mm10 reference genome, to compare transcript abundance of hormone receptors. Principal component analysis was performed using the prcomp function as implemented in the R stats package. Genes with a CPM expression value above 1 in at least 50% of the samples in a treatment group were included in statistical differential gene expression analysis (*n* = 8/group in OVX, E2, P4, and E2 + P4). Genes were determined as differentially expressed when fold change (FC) was above 2 (equal to a log2FC over 1) and false discovery rate (FDR) below 0.05. Gene ontology analysis was performed in ShinyGO v0.741 [40] using a background of all protein-coding genes.

### Western Blot

DSS-treated distal colon samples from experiment 1 were analysed for protein expression of ERα, IDO1, COX2, and ITLN1 by Western blot. Lysis and protein extraction were performed using the Nucleospin RNA and protein kit (Macherey-Nagel) and an Ultra-Turrax homogenizer (IKA). Extracted total protein was quantified using Pierce™ BCA Protein Assay Kit (Thermo Scientific™), and the protein concentration of all samples was diluted to equal amounts in PBS with final concentrations of 22.5% Laemmli sample buffer (Bio-Rad) and 2.5% β-mercaptoethanol (Sigma). Color Prestained Protein Standard, Broad Range (10–250 kDa, New England Biolabs) was used as a molecular weight indicator. After SDS-PAGE, the protein was transferred to methanol-activated PVDF membranes. Western blot was performed using 2% bovine serum albumin (BSA) for blocking and all antibody dilutions. Antibodies and dilutions provided in Supplemental Material 2. Signal was visualized using SuperSignal™ West Pico PLUS Chemiluminescent Substrate (Thermo Scientific™) and imaged with Sapphire biomolecular imager (Azure Biosystems). The signal was quantified using FIJI [41] and normalized to either GAPDH or ACTINβ within each sample.

### Hematoxylin-Eosin and Immunostaining

Longitudinal middle and distal colon samples were opened, cleaned from feces, and fixed in 4% formalin for 24 h before either paraffin embedding (FFPE, experiment 1) or frozen embedded in O.C.T. Compound (Tissue-Tek^®^) (one mouse from each treatment, experiment 2) and cutting. The uterus and skeletal muscle were sampled as FFPE. FFPE sections were deparaffinized and rehydrated before hematoxylin-eosin or immunostaining. Frozen sections were stepwise thawed and fixed using cold PFA 4% in PBS for 30 min at 4 °C before hematoxylin-eosin or immunostaining. No antigen retrieval was performed for frozen sections. Heat-induced antigen retrieval for FFPE sections (IF staining of ERβ, ITLN1, tPA, and uPAR and IHC staining of ERα, ERβ, GPER, and IDO1) was performed in a microwave in either Na-citrate, 0.1% Tween-20, pH 6 for 20 min or Tris-EDTA, pH 9 for 15 min, according to the manufacturer´s protocol for each antibody, or according to our own optimization if protocols for antigen retrieval were not provided. All samples were permeabilized using 0.5% Triton X-100 and blocked for 1 h at RT using 22.5 mg/ml glycine (Sigma-Aldrich), 5% normal goat serum (Merck) in 2% BSA in PBS. Primary antibodies were incubated overnight at 4 °C, and secondary antibodies for 1 h at RT.

For IF, the Vector^®^ TrueVIEW^®^ Autofluorescence Quenching Kit was used (5 min at RT), before mounting with VECTASHIELD Vibrance^®^ Antifade Mounting Medium with DAPI, both from Vector Laboratories. For IHC, sections were incubated in 0.3% hydrogen peroxidase/PBS between the first and second antibody staining to block endogenous peroxidase. DAB Substrate Kit, Peroxidase (HRP), with Nickel (VectorLaboratories) was used to detect HRP, and Mayer´s hematoxylin was used to counterstain the tissue. IHC samples were mounted using Aquatex^®^ mounting media (Sigma).

All antigen retrieval methods, antibodies, and dilutions are listed in Supplemental Material 2. The antibody signal was visualized using a Pannoramic Midi fluorescent scanner with a 20x objective (channels for DAPI, FITC, TRITC, and Cy5) and SlideViewer software (3DHISTECH Ltd.). Pseudo coloring was used and is described in the figure legends. Brightness, contrast, and gamma adjustments were performed in CorelDRAW 2025 software, version 26 (Corel Corporation).

### Antibody Validation and Controls

In addition to target validation by the manufacturer, antibodies used for staining estrogen receptors were verified in-house by Western blot was performed with ERα and ERβ antibodies and ER-positive (uterus, vagina) and -negative (skeletal muscle) SDS-PAGE separated mouse tissue lysates, which were gathered from healthy female C57BL/6 mice. The membranes were stripped using 0.2 mM glycine, 0.1% SDS, and 1% Tween-20 (pH 2.2) after capturing ERα and GAPDH signals. After this, the membranes were re-blocked and incubated with the ERβ antibody. Also, IHC staining of ERα, ERβ, and GPER in the mouse vagina (from experiment 1) was used for positive control and IF staining of skeletal muscle from healthy female C57BL/6 mice for negative or low expression tissue control. Rabbit IgG or secondary antibody-stained IF sections of the distal colon were used as negative antibody controls, produced together with the target staining, and imaged simultaneously with the same settings for exposure.

### Imaging Mass Cytometry

Cryosectioned longitudinal middle and distal colon samples from the first experiment were analysed using imaging mass cytometry (IMC). Sections were processed and stained as described above for IF, except that blocking was performed using 3% BSA in RT for 1 h. All primary antibodies were incubated overnight at 4 °C, and dilutions are listed in Supplemental Material 2. For DNA detection, Cell-ID Intercalator-Ir was incubated for 30 min at RT, and samples were dried. Images were acquired using a Hyperion Imaging System, and MCD Viewer was used for visualization (all from Standard Biotools). Pseudo colouring was used and is described in the figure legends. Brightness, contrast, and gamma adjustments were performed in CorelDRAW 2025 software, version 26 (Corel Corporation).

### Statistical Analysis

Statistical power for analysis of variance (ANOVA) testing was calculated using the G*Power software [42]. Calculations were based on the previously published total colitis scores [20], assuming unequal variances and with a two-tailed p value. The required sample size per group was calculated to be 5, with statistical power determined to be 0.99. Statistical analysis was performed in GraphPad v.9. Data was tested for normality of the distribution and equality of variances. Based on this, an appropriate parametric or non-parametric test was chosen. We used one-way ANOVA, two-way ANOVA, or mixed-effects analysis as parametric tests when variances were equal, Brown-Forsythe and Welch ANOVA for parametric testing when variances were unequal, and Kruskal-Wallis for non-parametric testing. For multiple comparisons, either Tukey´s, Dunnett´s, or Dunn´s post hoc tests were used as appropriate. In all figures, statistical significance is presented as asterisks, with * indicating p value 0.05 or under, ** 0.01 or under, *** 0.001 or under, **** 0.0001 or under. Correlation was calculated using Spearman’s R. The RNA sequencing dataset connected to the present study includes only genes with a false discovery rate less than 0.05.

## Results

### E2 Pre-treatment Exacerbated Colitis and Expanded Inflammatory Immune Populations, While OVX Protected From Inflammation

**Fig. 1:**
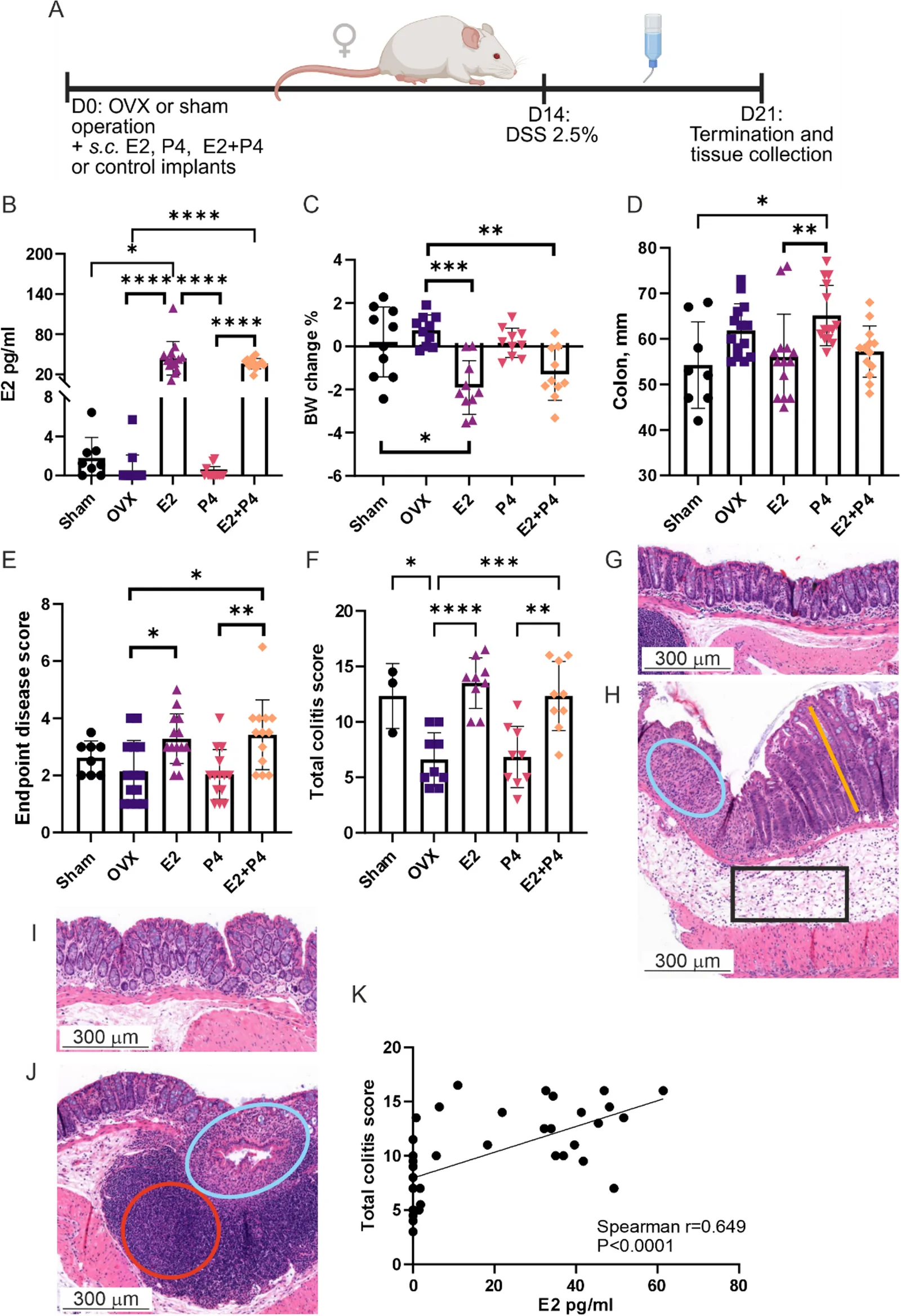
Ovariectomy protected from, while E2 exposure aggravated colitis. Mice were ovariectomized or sham-operated, and hormone (for E2, P4, E2 + P4 mice) or empty (for sham and OVX mice) pellets were implanted *s.c.* After three weeks, all mice were exposed to 2.5% DSS in drinking water for 7 days before termination and tissue collection (**A**, Image created in BioRender). Serum E2 concentration was significantly increased in E2-supplemented mice (**B**, pooled data from experiments 1 and 2). BW decreased during DSS induction of colitis in the E2-supplemented animals, compared to OVX (data pooled from two experiments, **C**). Colon length was decreased in the E2-exposed mice, compared to P4 (data pooled from two experiments, **D**). Endpoint disease activity scores (data pooled from two experiments, **E**), as well as total colitis score, which included the histological quantification of inflammation, were both increased in the E2-exposed mice compared to both OVX and P4 (pooled, **F**). **G**-**J** representative images from OVX, E2, P4, and E2 + P4, respectively. The inflammation-limiting effect of E2-depletion was apparent in the HE-stained distal colon sections of OVX and P4 mice, while in the E2-exposed mice, inflammation was represented by crypt elongation (H, orange line), ulcers with crypt loss (**H**, **J**, blue oval), extensive edema (**H**, black box), and extensive immune infiltration (**J**, red circle). Serum E2 levels correlated with total colitis score of all histologically scored mice (**K**, exp 1 *n* = 33 and exp 2 *n* = 5, Spearman r 0.65, 95% confidence interval 0.42 to 0.83). Data is presented as mean and standard deviation. Each data point represents a single animal; total number of animals: sham *n* = 8, OVX, E2, P4, E2 + P4 *n* = 14 in each group, except for histological analysis and total colitis score quantification, where sham *n* = 3, OVX, E2, P4, E2 + P4 *n* = 9 in each group. Statistical analyses with Kruskal-Wallis, and Dunn´s test for multiple comparison (**B**, **D**-**F**), or ordinary one-way ANOVA (**D**), together with Tukey´s test for multiple comparison

To examine the effect of ovarian hormones in colitis, young adult female FVB/n mice were ovariectomized, and a hormonal supplementation implant was administered subcutaneously for three weeks (E2, P4, E2 + P4, or empty pellets for sham-operated and OVX groups). After recovery, all mice received 2.5% DSS in drinking water (Fig. 1A). During the experiment, body weight (BW) and colitis symptoms were followed. The hormone effects were verified by measuring the serum levels of E2, P4, and E1 as well as by weighing and visually assessing HE-stained sections of the uterus. Previously, decreased uterus weight as a sign of hormone depletion has been reported as early as one week after OVX [43]. At termination, mean serum levels of E2 were decreased (Fig. 1B), and neither uterus weight nor appearance indicated any significant E2 effect in OVX and P4 mice (Supplemental Fig. 1 A and Bi-Bv). Hence, these groups are referred to as E2-depleted. The serum E2 concentration was increased in E2 and E2 + P4 (E2-supplemented) mice, compared to both sham and the E2-depleted mice (Fig. 1B). The increased weight and size of the uterus confirmed an E2 effect for the sham-operated and E2-supplemented mice (Supplemental Fig. 1 A and Bi-Bv); hence, these are together referred to as E2-exposed. As E2 can be converted to estrone (E1) [44], serum levels of E1 were also measured. The E2 and E2 + P4 supplementation resulted in serum concentrations of E1 exceeding those of the sham-operated mice, while the serum P4 was similar in sham, P4, and E2 + P4 mice (Supplemental Fig. 1C-D).

OVX induces weight gain, and as expected, the E2-depleted mice increased most in weight before the DSS challenge, while E2-supplemented mice increased weight modestly (Supplemental Fig. 1E). The E2-depleted mice, which increased the most in weight, were less severely affected by the induced colitis, while sham and E2-supplemented mice were the most inflamed (Fig. 1C-F). E2-exposure decreased BW and colon length, which are signs of intestinal inflammation (Fig. 1C-D) and exacerbated colitis symptoms, such as fecal softening and diarrhoea, together with intestinal bleeding (Supplemental Fig. 1F-G). The symptom scores were similar in both experiments (Supplemental Fig. 1H-I) and were combined with body weight loss into a disease score. At termination, the disease score was increased in the E2-exposed mice (Fig. 1E). The total colitis score, calculated from the symptom score combined with the histological signs of inflammation in the distal colon, was decreased in the E2-depleted mice (Fig. 1F). The proximal colon was excluded from this study, as DSS-induced inflammation affects the distal parts of the colon more strongly [19]. The differences in the histological signs of colitis, such as epithelial erosion (ulcers with total loss of crypt structures and all normal epithelial cell types), edema, immune infiltration, and epithelial hyperproliferation are represented in Fig. 1G-J, depicting sections from OVX, E2, P4, and E2 + P4 groups, respectively. Serum concentration of E2 correlated with the total disease score (Fig. 1K). Additionally, E1, but not P4, correlated with the total disease score (Supplemental Figure J-K).

Together, the results presented here indicate E2 as a significant inflammation-aggravating hormone when administered as a pre-treatment. Simultaneously, P4 did not affect inflammation alone, nor in combination with E2, in this time frame, and the E2 and E2 + P4 mice did not differ substantially from each other in levels of inflammation. This reflects the previously observed inflammatory effect of ERα, but not PGR, activation initiated before the induction of colitis [20, 21].

**Fig. 2:**
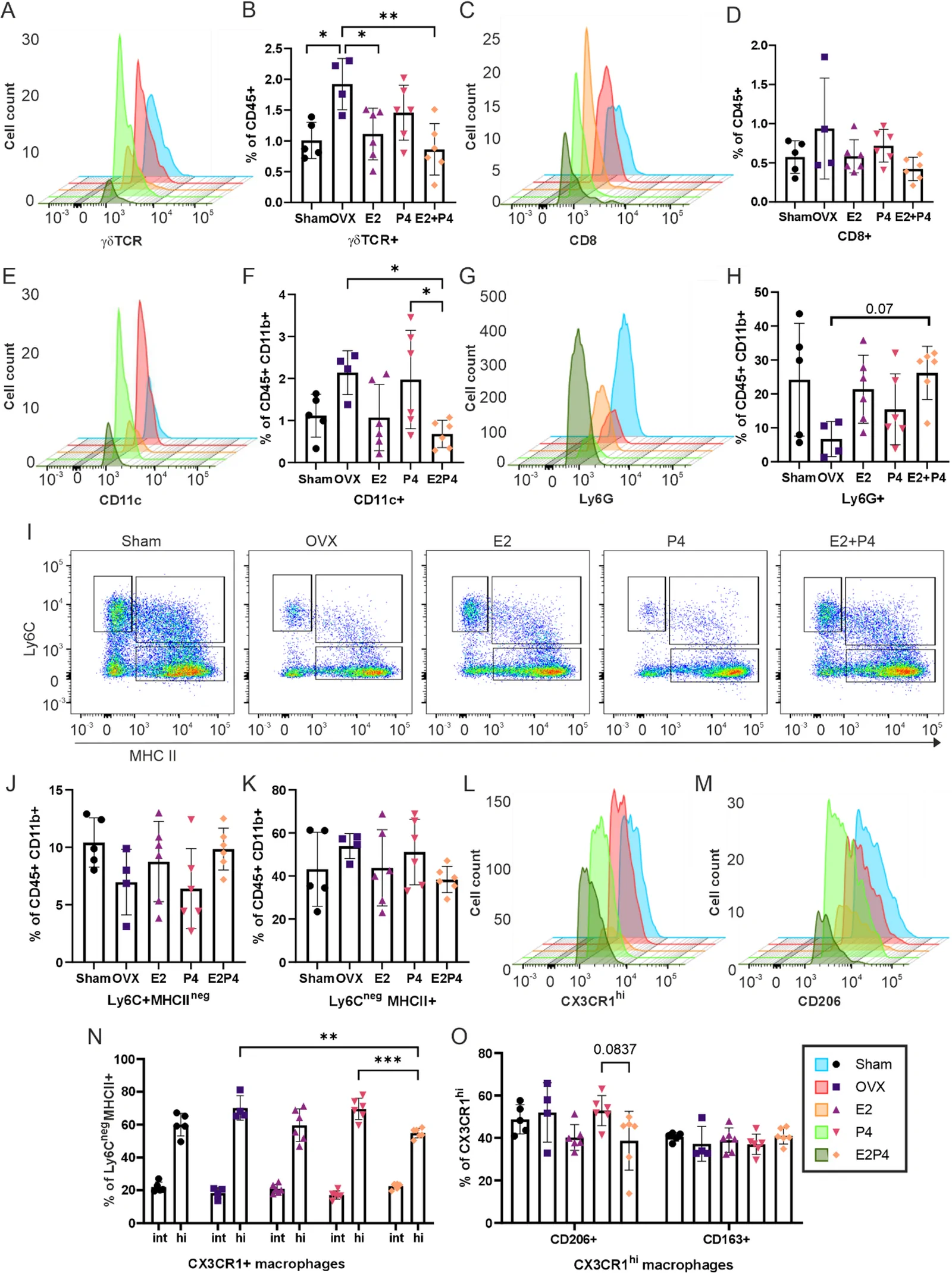
Anti-inflammatory immune cell populations were expanded in E2-depleted mice. The percentage of γδ T cells in the CD45 + cell population decreased in E2-aggravated inflammation, compared to OVX (**A**, **B**), and a similar pattern of CD8 + T cell alteration was observed (**C**, **D**). CD45 + CD11b+Ly6G^neg^CD11c + DCs decreased in E2 + P4-treated mice, compared to both OVX and P4 (**E**, **F**), while CD45 + CD11b+Ly6G+ neutrophils decreased in the CD45 + CD11b+ innate immune cell population of E2-depleted mice compared to OVX (**G**, **H**). Patterns of a decreased CD45 + CD11b+Ly6G^neg^CD11c^neg^Ly6C+MHCII^neg^ monocytes (**I**, **J**), together with increased CD45 + CD11b+Ly6G^neg^CD11c^neg^Ly6C^neg^MHCII+ macrophages (I, K) were observed in the CD45 + CD11b+ population of E2-depleted mice. Also, the mature CD45 + CD11b+Ly6G^neg^CD11c^neg^Ly6C^neg^MHCII+F480 + CX3CR^hi,^ but not CX3CR^int^, macrophages increased in E2-depleted mice compared to E2P4 (L, N), and among these CX3CR^hi^ macrophages, the amount of CD206 + anti-inflammatory maker was almost statistically significantly increased in P4 mice, compared to E2P4 (M, O), while CD163 + macrophages were not extensively altered (N-O). A, C, E, G, J, L, and M are representative images, showing samples close to the mean value of each treatment group, with the color code in the lower-right corner. Data is presented as mean and standard deviation, sham *n* = 5, OVX *n* = 4, and E2, P4, E2 + P4 *n* = 6 in each group. Statistical analysis was performed either using one-way ANOVA (B, D, F, H, J, K) or Two-way ANOVA (N, O), with Tukey´s test for multiple comparisons

Flow cytometry was used to investigate the E2-induced alterations in immune cell populations. CD45, the leukocyte common antigen, was used as a marker to select all immune cells, and integrin CD11b was used to select innate immune cells. CD45 + γδTCR + T cells, which display innate immune cell-like qualities, were increased in OVX mice, compared to E2 (Fig. 2A-B, see also the gating strategy for the whole analysis in Supplemental Fig. 2 A). Although cytotoxic T cells have been associated with colitis, a statistically nonsignificant pattern of increased CD45 + CD8+ T cells was observed in E2-depleted mice (Fig. 2C-D). Monocyte-derived CD45 + CD11b+Ly6G^neg^CD11c+ dendritic cells (DCs), which were MHCII^hi^ (mean 93.8%, SD 5.3%), are important antigen-presenting cells and play a role in lymphocyte activation [45]. CD11c + DCs were increased in E2-depleted mice (Fig. 2E-F), indicating either decreased numbers or migration to mesenteric lymph nodes.

As a sign of the ongoing acute inflammation in E2 + P4 mice, the mean percentage of Ly6G+ neutrophils in the CD45 + CD11c+ innate immune cell population was higher compared to OVX, although not statistically significant (Fig. 2G-H). In health, the largest population of macrophages in the colon is derived from circulatory Ly6C+ monocytes [46]. These mononuclear phagocytes differentiate through intermediate states, finally maturing into CX3CR1 + CD206+ IL-10-secreting subepithelial macrophages [47]. Differentiation of monocytes to macrophages is characterized by decreasing Ly6C and increasing MHCII expression. In inflammation, the amount of infiltrating Ly6C+ monocytes increases, while the fraction of mature, tolerance-inducing CX3CR1 + and CD206 + macrophages decreases [48]. Here, opposing patterns of decreased Ly6C+MHCII^neg^ monocyte-derived cells together with an increase in the percentage of Ly6C^neg^MHCII+ monocyte-derived macrophages were observed in E2-depleted mice (Fig. 2I-K). Additionally, reflecting the protective effect of E2-depletion, the percentage of mature MHCII+F480 + CX3CR1 high (hi), but not CX3CR1 intermediate (int), expressing macrophages was decreased in E2 + P4-treated mice, together with a decrease in the percentage of scavenger receptor CD206 + cells within the MHCII+F480 + CX3CR1^hi^ macrophage population (Fig. 2L-O). Even though the expression of another scavenger receptor, CD163, has also been associated with anti-inflammatory function in mucosal macrophages [48], the percentage of MHCII+F480 + CX3CR1^hi^CD163+ macrophages did not alter by treatment (Fig. 2O, Supplemental Fig. 2B). To summarize, inflammatory innate immune cell populations, such as monocytes and neutrophils, were reduced in the colons of E2-depleted mice, consistent with the reduction of disease score and the histological signs of inflammation. Simultaneously, an increase of homeostatic immune cells, such as γδTCR + T cells and mature CX3CR1^hi^ CD206 + macrophages, was observed. In the healthy colon, these cells function in the surveillance of the commensal microbiota, which is important for maintaining homeostasis [49, 50].

### Estrogen Receptors Were Expressed in Different Tissue Components of the Colon

**Fig. 3:**
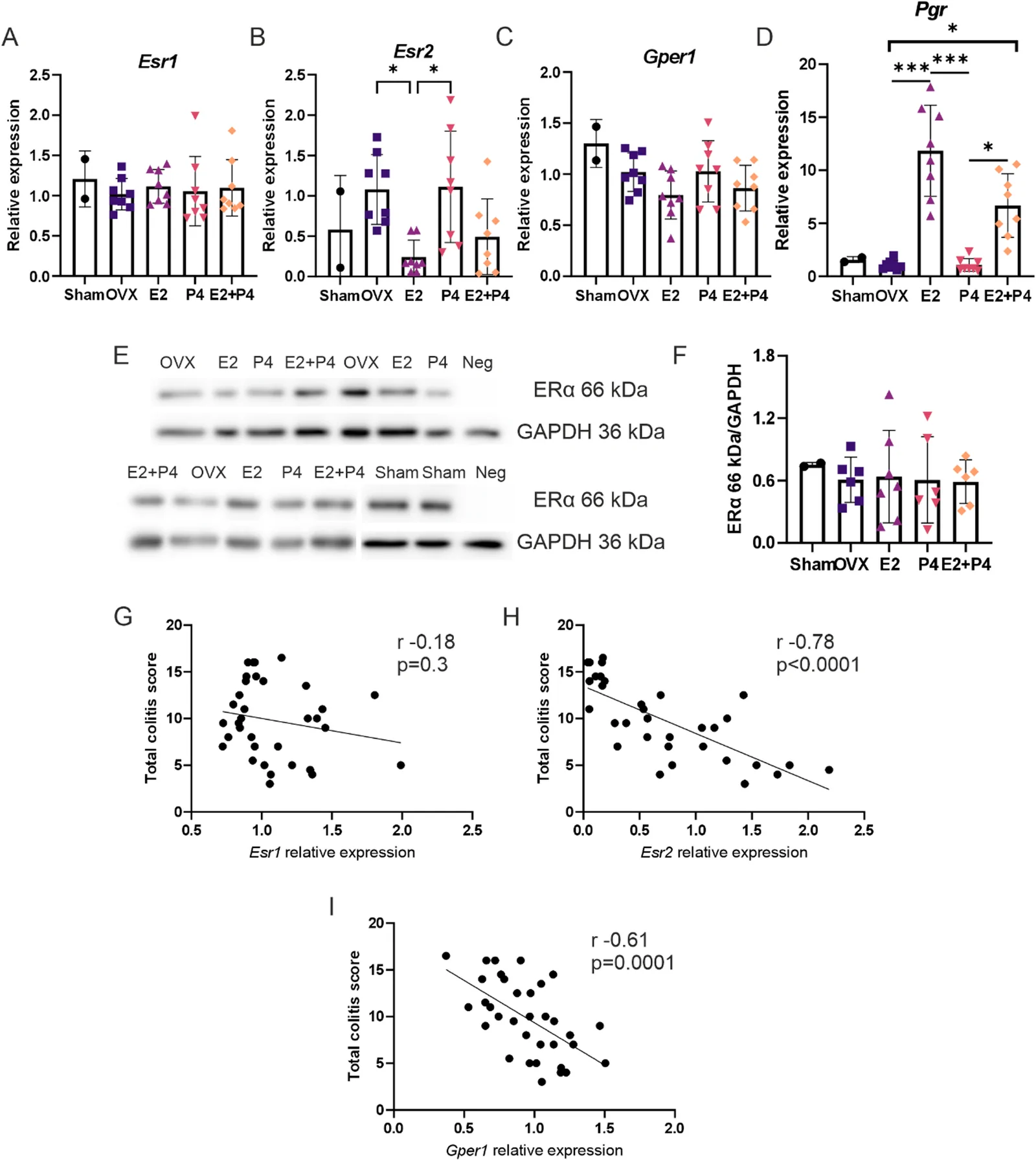
Estrogen receptor expression in the distal colon. *Esr1* was expressed equally in all mice (**A**), while *Esr2* (**B**) and *Gper1* (**C**) were expressed in similar patterns, but with a statistically significant decrease only for *Esr2* expression in E2 mice. *Pgr* was upregulated in E2-supplemented mice (**D**). Sham *n* = 2, OVX, E2, P4, E2 + P4 *n* = 8/group, experiment 1. No significant differences were seen in ERα protein between the groups when protein levels were analyzed by Western blotting using mouse skeletal muscle as a negative control (**E**-**F**, sham *n* = 2, OVX, P4, E2 + P4 *n* = 6, and E2 *n* = 7, experiment 1). Total colitis score did not correlate with RT-qPCR measured relative expression of *Esr1*, while both *Esr2* and *Gper1* expression negatively correlated with inflammation scores (**G**-**I**). Data is presented as mean and standard deviation, sham *n* = 2, OVX, E2, P4, E2 + P4 *n* = 8/group. Statistical analysis using one-way ANOVA and Tukey´s test for multiple comparisons (**A**-**D**, **F**). Correlation of estrogen receptors and inflammation was analyzed using Spearman´s non-parametric test (*n* = 34)

RT-qPCR was used to analyze potential differences in hormone receptor levels in the distal colon. *Esr1* transcripts were found in equal amounts through the treatment groups (Fig. 3A, Supplemental Fig. 3 A). Consistent with the previous reports [20, 22] *Esr2* was downregulated in the inflamed, E2-supplemented mice, compared to the non-inflamed, E2-depleted mice (Fig. 3B, Supplemental Fig. 3B). *Gper1* displayed a similar expression pattern (Fig. 3C, Supplemental Fig. 3 C). *Pgr* expression, which is upregulated by ERα activation [51], was highly increased in the E2-supplemented mice (Fig. 3D, Supplemental Fig. 3D). As *Esr2* and *Gper1* were downregulated in E2-exposed mice, the estrogen receptor expression correlation to inflammation was analyzed. The expression pattern between treatment groups in the whole colon samples (Fig. 3A-D, experiment 1) was similar to that of the digested and strained distal colon cell population, which was also used in cytometry (Supplemental Fig. 3A-D, experiment 2). In general, the expression levels of the estrogen receptors in the distal colon were low. Out of the different estrogen receptors, *Esr1* transcripts were the most abundant, and *Esr2* or *Gper1* the least abundant by RT-qPCR Cq and RNAseq TPM (Supplemental Fig. 3E-F). A slight discrepancy was observed between *Esr2* expression levels in the two different sample types analyzed (Supplemental Fig. 3E). *Pgr* expression in the distal colon was even lower compared to the estrogen receptors, except for the E2-supplemented mice. *Pgr* was also more highly expressed in the whole-colon samples, possibly indicating that the abundance of ERα activation depends on the sample composition (Supplemental Fig. 3E). Distal colon ERα protein levels were found to resemble the *Esr1* expression, and no treatment differences were observed (Fig. 3E-F, representative membranes in Supplemental Fig. 3G). There was no correlation observed between *Esr1* expression (r −0.18, *p* = 0.3), while *Esr2* (r −0.78, *p* < 0.0001) and *Gper1* (r −0.61, *p* = 0.0001) both negatively correlated with the total colitis score (Fig. 3G-I). These results indicate a functional role for ERα, more dependent on activation, while ERβ and GPER are downregulated in inflammation.

**Fig. 4:**
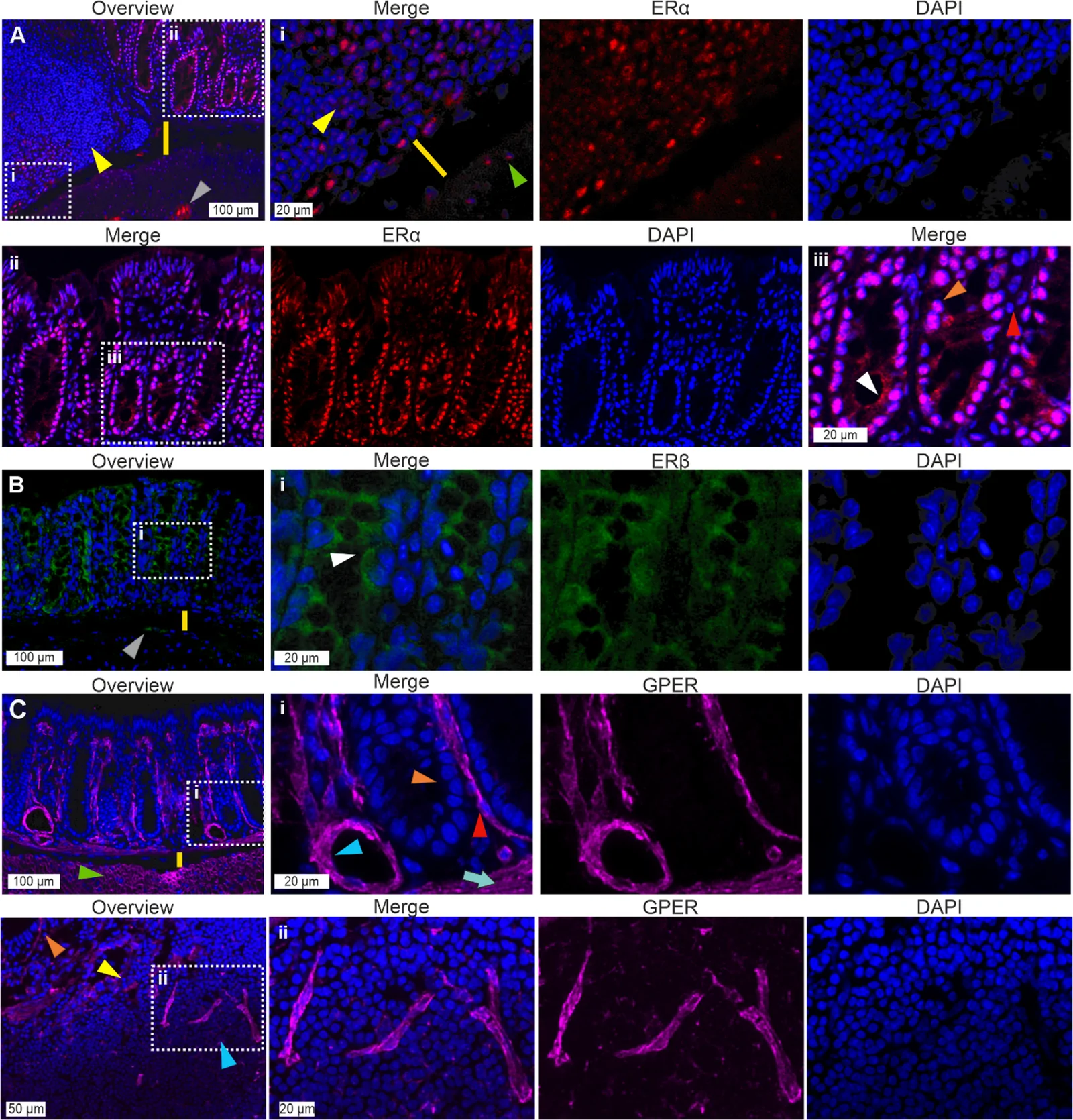
Estrogen receptor localization in the distal colon. ERα was observed in epithelial cells, in muscularis mucosa and in GALT (A, overview and magnified in i and ii), as well as in smooth muscle and Auerbach´s plexus (A, lower panel). ERα localized to the cytoplasm and nucleus in colonocytes, as indicated by the red and blue colors converting to purple, but not in intercrypt LP cells (Aiii). ERβ was localized to the epithelia and the enteric nervous system (B, overview and magnified in i). The GPER (pseudo colored magenta) was observed in intercrypt LP cells, the muscularis, and in submucosal vessel structures, but not in colonocytes (C, overview and magnified in i). GPER was also expressed in vessel structures in the distal colon GALT (C, lower panel and magnified in ii). A-C are representative pictures, ERα and GPER staining carried out on 1 sample/treatment and ERβ sham *n* = 2, OVX *n* = 2, E2 *n* = 1, P4 *n* = 3, E2 + P4 *n* = 2, with similar signal patterns in each sample. Annotations indicate the following: orange line - edema, orange arrowhead – crypt colonocytes; yellow arrowhead – GALT; white arrowhead – colonocyte cytoplasm; red arrowhead – intercrypt LP cells; light blue arrowhead – vessels, green arrowhead – muscularis propria; gray arrowhead - enteric plexus; light blue arrow – muscularis mucosae

After quantifying levels, the localization of estrogen receptors in the mouse distal colon was determined by immunofluorescent staining. Even though *Pgr* was found at least as a low-level transcript in the colon, PGR was excluded from the immunostainings, as the effect of P4 in this colitis model was negligible (Fig. 1). ERα was observed in the cytoplasm and nuclei of epithelial cells throughout the crypt and in gut-associated lymphoid tissue (GALT), as well as in the enteric nervous system and smooth muscle of the intestinal wall (Fig. 4Ai-iii). Some similarities were observed between the different receptor subtype localizations. ERβ was also expressed in the colonic epithelium as well as in the enteric nervous system, but not in cells with immune morphology (Fig. 4B). Similar to ERα, GPER was observed in smooth muscle (Fig. 4A and C). However, GPER was not observed in epithelial cells, but instead, in the intercrypt *lamina propria* (LP), as well as in individual cells and vessel structures of the mucosa, submucosa (Fig. 3C, upper panel), and gut-associated lymphoid tissue GALT (Fig. 3C, lower panel). No differences in receptor expression patterns were observed between the groups, other than those related to the amount of immune infiltration or crypt loss presented in Figs. 1 and 2. Specific binding of antibodies was verified by Western blotting of ERα and ERβ positive and negative tissue samples from mice (Supplemental Fig. 4A-D), as well as IHC staining of ERα, ERβ, and GPER positive mouse vagina and IF staining in skeletal muscle, which was negative for ERα, but exhibited low expression of ERβ and GPER (Supplemental Fig. 4E-F). Images of negative control staining of the distal colon are provided in Supplemental Fig. 4G-J.

### ERα was Coexpressed in CD206+, CD3+, and CD19 + Immune Cells

As the aim was to understand the drivers of E2-aggravated inflammation, we used explorative IMC and qualitative IF to visualize estrogen receptors in different immune cells in the distal colon. As ERβ was not observed in cells with immune morphology (Fig. 4B), only ERα and GPER were examined by immunostaining receptors together with commonly used immune markers. Similarly to the flow cytometry analysis, the leukocyte common antigen CD45 was used as a marker to detect all immune cells, and the integrin CD11b to detect innate immune cells. GPER expression was analyzed using IMC of cells within the submucosal edema (Supplemental Fig. 5 A). We observed unidentified cells that did not express immune markers CD45 or CD11b, with strong nuclear or perinuclear GPER staining in the vicinity of GPER+CD45^low^CD11b+ cells (Supplemental Fig. 5B). The GPER+CD45^low^CD11b+ cells expressed monocyte/macrophage marker F4/80, but not CD80, which is associated with immune activation (Supplemental Fig. 5 C and D). Further, the GPER+F4/80 + also expressed mature macrophage marker CD206 (Supplemental Fig. 5E).

**Fig. 5:**
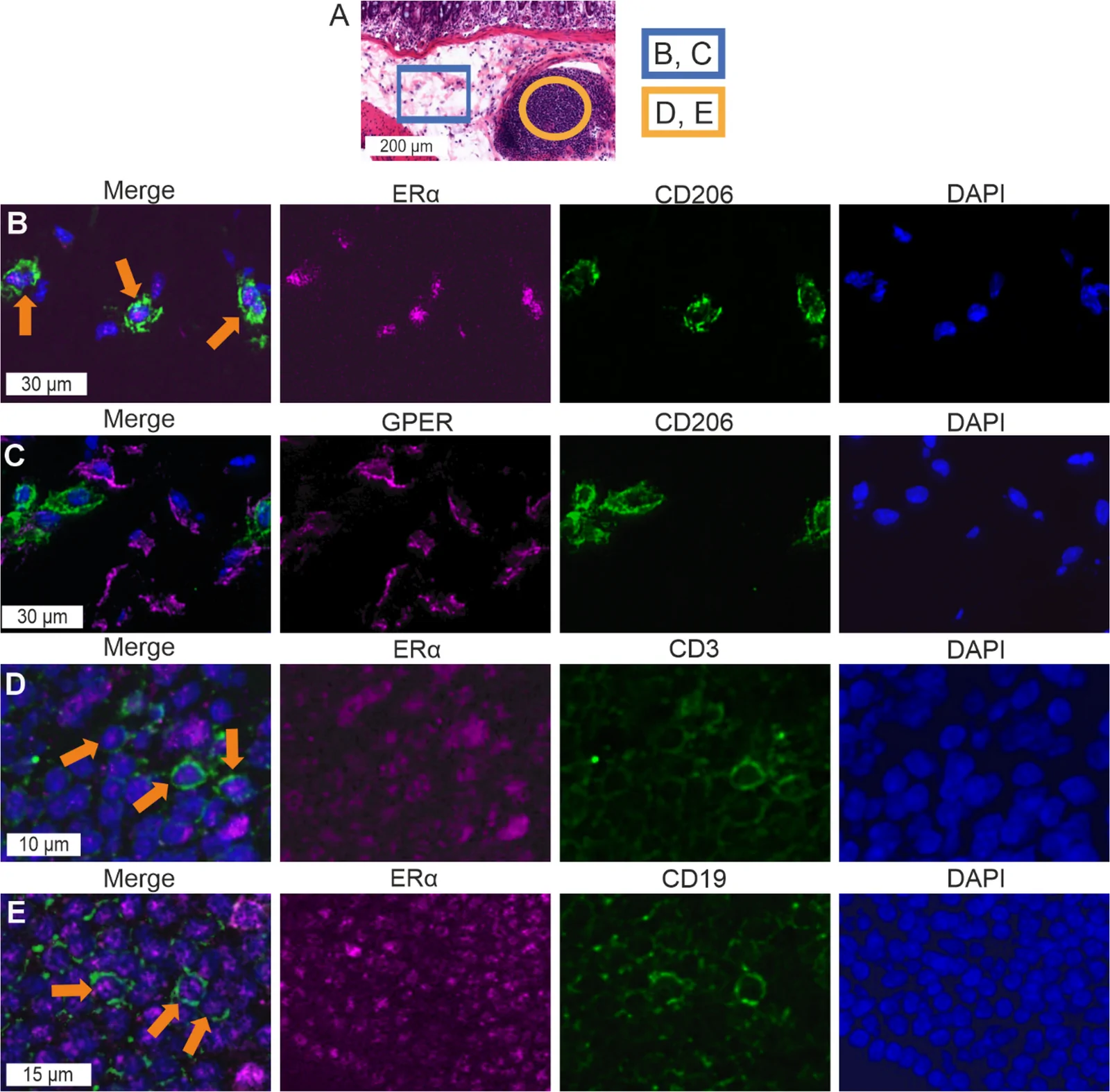
Estrogen receptors were observed in innate and adaptive immune cells in the distal colon by IF. Representative overview image indicating the tissue localization of the higher magnifications in B-E. Blue box indicates submucosal edema, and the orange circle indicates GALT (**A**). Nuclear ERα was observed in CD206+ (**B**) cells, while cytoplasmic GPER was observed in unidentified cells, but not in CD206 + macrophages (**C**). ERα was also observed in the nuclei of CD3 + T cells (**D**) and CD19 + B cells (**E**). Antibody signals (Alexa647) were pseudo-colored as magenta for ERα (**B**) and GPER (**C**), and green (Alexa568) for CD3 (**D**) and CD19 (**E**). Orange arrows indicate cells with coexpression of ERα with the respective immune cell marker within the same cell (B, D, E). Each staining was repeated in 5 different mice for ERα + CD206, GPER+CD206, and 4 mice for ERα + CD3 and ERα + CD19, *n* = 1/treatment group

Apart from the data shown in Fig. 4, ERα was also localized in the nucleus of cells scattered throughout the epithelia and submucosal edema. Thus, further investigation of the coexpression of estrogen receptors together with immune markers was carried out using IF. Here, the mannose receptor CD206 antibody was used to detect mature monocyte-derived macrophages, the T cell coreceptor CD3 was used to detect T cells, and the B cell coreceptor CD19 was used to detect B cells in the distal colon, of which a representative overview image is provided in Fig. 5A. Figures 5B-C depicts submucosal edema, and Figs. 5D-E represent GALT. This analysis revealed that ERα was present in the nuclei of CD206 + macrophages (Fig. 5B). However, contrary to the IMC results, CD206 + macrophages did not express observable amounts of GPER, while the adjacent, previously unidentified cells (Supplemental Fig. 5) were clearly GPER+ (Fig. 5C). This might indicate, for instance, that the GPER signal in the mature macrophages was lower and only detectable with the more sensitive IMC method [52], but further study is needed to verify this. Further, ERα was also detected in the nucleus of CD3 + T cells, as well as in CD19 + B cells (Fig. 5D-E). These findings indicate that multiple cell types involved in the inflammatory response in the colon are indeed estrogen-responsive, and that ERα is most likely involved in the inflammatory signaling.

### E2 Pre-Treatment Upregulated Genes Associated With the Epithelial Antimicrobial Response and Innate Immune Cell Activation, and Downregulated Genes Related to Metabolic Pathways

**Fig. 6:**
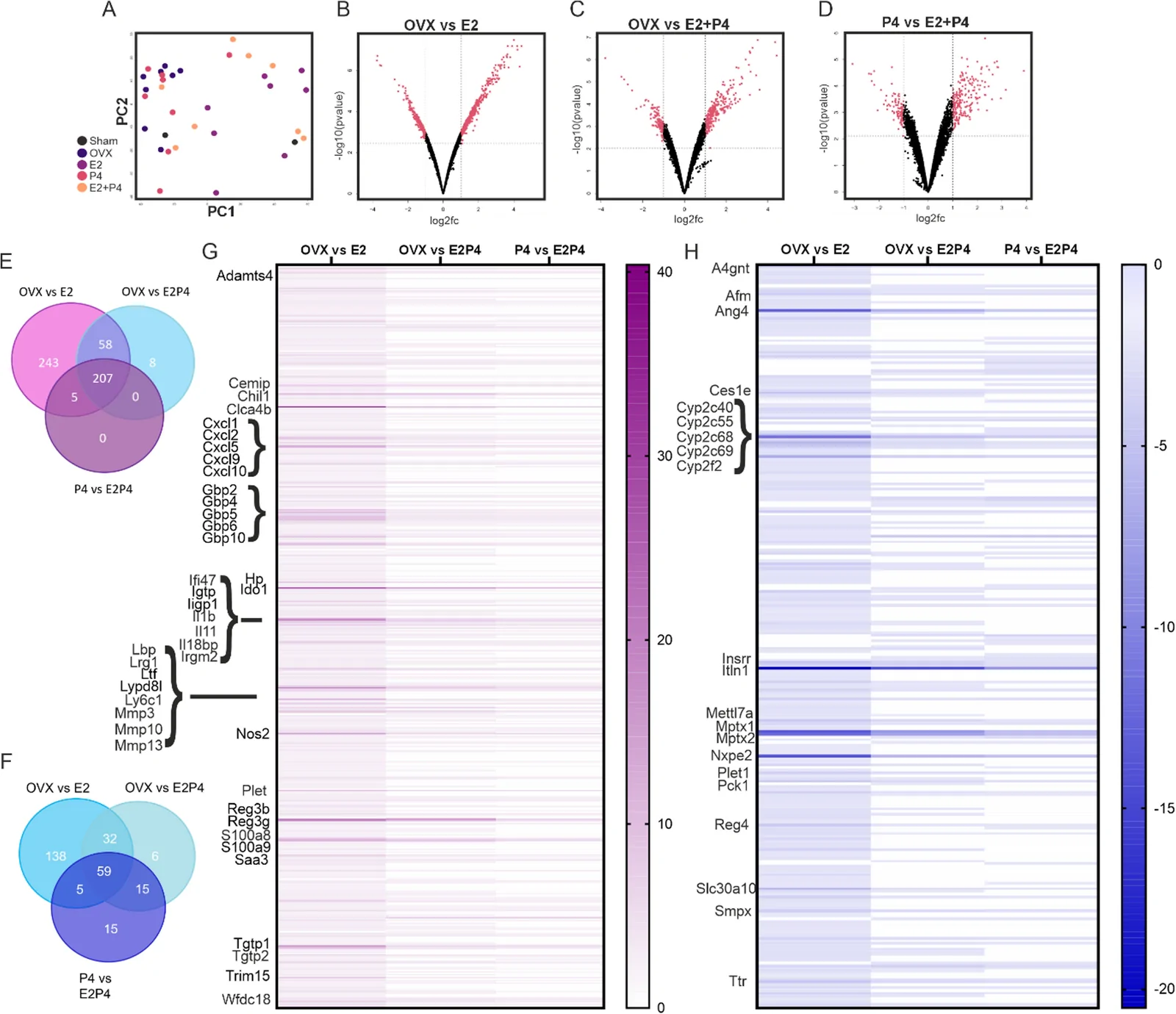
Differentially expressed genes in E2 aggravated colitis. PCA of distal colon bulk RNAseq suggested inflammation to be the main determinant of gene expression in the samples (**A**). A total of 791 genes were differentially regulated among the treatment groups. In the comparison between OVX and E2, 521 genes were upregulated, and 270 genes were downregulated (**B**). In the comparison between OVX and E2 + P4, 272 genes were upregulated and 112 downregulated (**C**), while 212 upregulated and 94 downregulated genes were found in the P4 and E2 + P4 comparison (**D**). Upregulated DEGs shared or unique to treatment groups (**E**). Downregulated DEGs shared or unique to treatment groups (**F**). Among the upregulated genes, 42 were upregulated more than 7-fold in E2-supplemented mice, compared to OVX (**G**, color scale indicates fold changes), and 21 genes were downregulated more than − 4-fold (**H**). Two sham mice were included in RNAseq. OVX, E2, P4, E2 + P4 mice were analyzed for differentially expressed genes, all *n* = 8

To identify genes or signaling pathways that define the mechanism of the E2 inflammatory effect, we sequenced total mRNA from the distal colon. Differentially expressed genes (DEGs) were analyzed by comparing expression between E2-supplemented and -depleted mice. The OVX vs. E2, OVX vs. E2 + P4, and P4 vs. E2 + P4 comparisons yielded statistically significant DEGs. Principal component analysis (PCA) of gene expression indicated that E2-depleted mice differed from the E2-supplemented, with some exceptions (Fig. 6A). A total of 791 DEGs were observed in the comparisons between the treatment groups, and the most pronounced alterations were seen in the E2-supplemented mice. The analysis found 747 DEGs in E2 mice compared to OVX, of which 521 genes were upregulated, and 270 genes were downregulated (Fig. 6B). In the animals supplemented with E2 + P4, the number of DEGs was lower, with 272 upregulated and 112 downregulated genes compared to the OVX group (Fig. 6C), and 212 upregulated and 94 downregulated genes compared to the P4-supplemented animals (Fig. 6D). Of the upregulated DEGs, 207 were common to all comparisons, while 243 DEGs were unique to the E2 vs. OVX comparison (Fig. 6E). Of the downregulated DEGs, 59 were shared between the comparisons, while 138 were unique to the OVX vs. E2 comparison (Fig. 6F). Among the upregulated genes, 42 were altered more than 7-fold in the E2-supplemented mice (Fig. 6G). Among the downregulated genes, 21 were altered more than 4-fold (Fig. 6H). The fold-change cutoffs were determined for a clear visualization.

Overall, the DEGs provided insight into the inflammatory processes in the colon. DSS is known to disrupt the mucus barrier, allowing bacterial colonization of the inner layer, which induces immune activation [53]. This was strongly reflected in the DEGs of the E2-supplemented mice. Upregulated epithelial anti-microbial response genes included *Ido1*,* Reg3*, Hp,* Chil1*,* Nos2*,* Ltf*,* Tgtp*,* Lypd8l* and *Pla2ga2*. Simultaneously, genes associated with epithelial stem cells (*Plet1*,* Lgr5*) were downregulated as colonocyte differentiation increases in response to colitis, also seen in these results as crypt elongation (Fig. 1H). Additionally, goblet cell-associated *Best2* and *Itln1* were downregulated, and fibroblast-associated genes, such as *Pfkfb3*,* Ccn5*, *Il33*, *Il11*, and *Saa3*, were upregulated.

Also, E2-supplementation substantially increased signaling through pathways associated with innate immune function and activation. Upregulation of the interferon response *Gpb* and *Ifi* family members was observed, together with increased expression of inducible inflammatory signal transducers like *Cox2* and *Nos2*. Chemoattraction and migration-associated genes, such as interleukins and inflammasome-associated NOD-like receptors, *Ccl* and *Cxcl* families, integrins, and matrix proteinases, were upregulated as well. Although individual variance decreased the statistical significance of the increased neutrophil influx in E2-exposed mice (Fig. 2H), the DEGs indicated increased neutrophil numbers and activity, as the *Ly6g* neutrophil marker and the subunits of the IBD biomarker calprotectin genes were upregulated. Also, upregulation of inflammatory monocyte-associated *Ly6c*,* Trem1*, and *Il1b* [47] supported the observation of increased monocyte influx and activity in the E2-exposed mice, even though the Ly6C+ cytometry results did not reach statistical significance (Fig. 2J). Additionally, *Siglec1* (CD169), *Ccl7*, and *Ccl8* were upregulated in E2 mice, reflecting an increase in intestinal damage-sensing macrophages, which recruit inflammatory monocytes from circulation [54]. Although both CD206 and CD163 are commonly used as markers of anti-inflammatory activity in macrophages [48], the gene expression data indicated a modest increase in *Cd163* expression, even though the CX3CR1^hi^CD163 + macrophages were not altered (Supplemental Fig. B). This might reflect increased bleeding and hemoglobin scavenging in inflammation, as heme induces oxidative stress and is therefore degraded by macrophages [55]. Interestingly, even though most DEGs in this dataset were related to innate immunity, B cell expansion and activation-associated genes such as *Cd19*,* Cd22*,* Cd37*,* Cd79*, and *Serpina9* were downregulated in the E2-supplemented, inflamed mice.

However, inflammatory gene expression might also be activated secondary to the E2-ERα direct transcriptional effect, as a part of the inflammatory cascade. Thus, ERE-regulated genes were identified from the dataset. In the E2-supplemented mice, we observed upregulation of *Pgr*,* Il6*,* Igfbp4*,* Ptges*,* Serpine1*,* F10*,* Plat*,* Plau*,* Plaur*,* C3*,* Lmcd1*, and *Thbs1*, as well as downregulation of *Esr2*,* Cyp1a1* and *Rapgefl1* [56–59]. The complete list of statistically significant DEGs is available in Supplemental Material 1.

The distal colon protein level expression of genes found in the RNAseq was verified by Western blot and immunostaining. IDO1, which was among the strongest upregulated genes in the dataset, was observed specific to the epithelium of both OVX and E2-supplemented mice (Supplemental Fig. 6A-B). Western blot confirmed protein levels of IDO1 up to 10-fold upregulated in E2 mice (Supplemental Fig. 6C-D). COX2, which produces prostaglandin E2 from arachidonic acid and is linked to estrogen signaling [60], increased in E2-supplemented mice, as the RNAseq data indicated (Supplemental Fig. 6E-F). Additionally, increased expression of ITLN1, which was the strongest downregulated gene in our E2 dataset, was observed in OVX mice (Supplemental Fig. 6G-H). ITLN1 was expressed in intact luminal epithelium and in goblet cells, while the expression was weak in areas with epithelial erosion, where crypt structures were lost (Supplemental Fig. 6I yellow box, Supplemental Fig. 6J-K). Additionally, the localization of products of directly ERα-regulated *Plat* and *Plaur*, whose products are tissue-type plasminogen activator (tPA) and plasminogen activator urokinase receptor (uPAR), were investigated. In the distal colon, these were observed in cells with immune morphology located in the submucosal edema (Supplemental Fig. 6I black box, Supplemental Fig. 6L-M), which was more prevalent in the E2-supplemented mice (Fig. 1F-J). Here, the monocyte and macrophage common marker F4/80 was used to elucidate tPA and uPAR expression in immune cells. While a strong tPA signal was observed in F4/80 + cells with rounded nuclei, uPAR signal was observed to be stronger in neutrophils, recognizable by the segmented nucleus (Supplemental Fig. 6L-M).

**Fig. 7:**
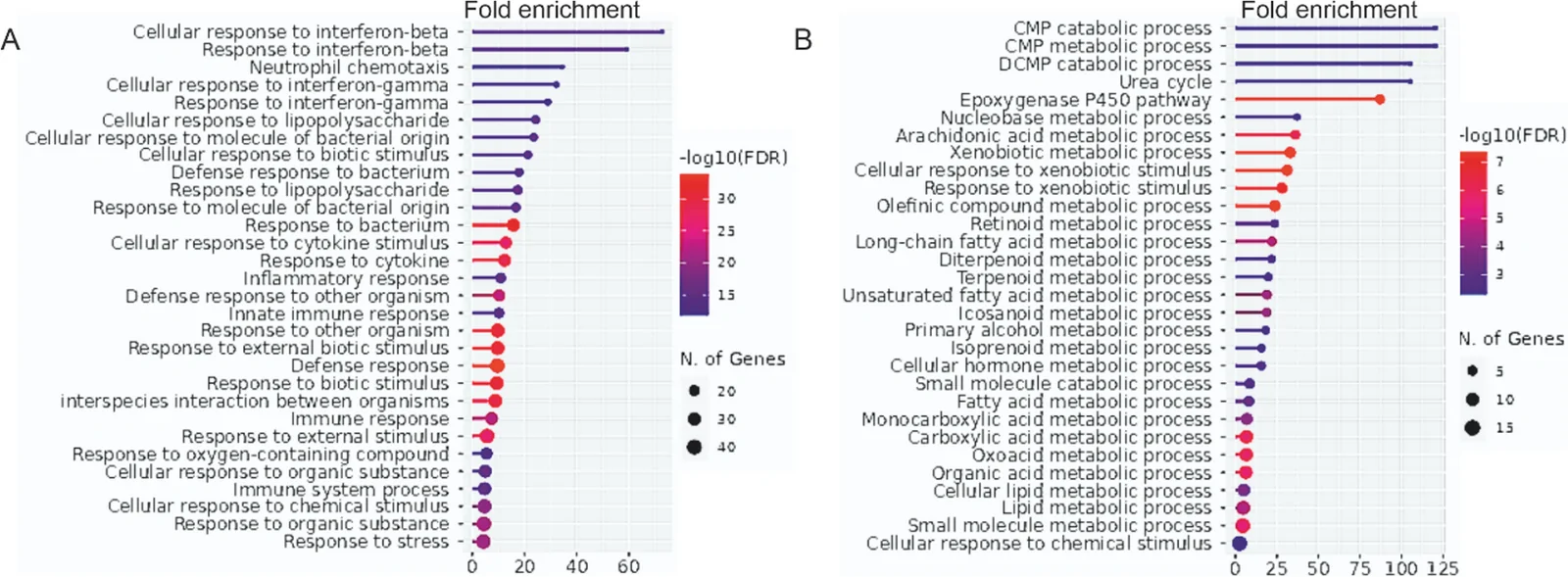
Gene ontology analysis of the differentially expressed genes in the distal colon. GO terms of the upregulated genes common to both E2-supplemented groups compared to OVX (**A**) and GO terms of the downregulated genes, common to all comparisons between E2-depleted and E2-supplemented mice (**B**)

Gene ontology (GO) analysis was performed based on DEGs from the comparisons of non-inflamed E2-depleted mice (OVX) and inflamed, E2-supplemented mice (OVX vs. E2, OVX vs. E2 + P4, and P4 vs. E2 + P4). The most enriched pathways observed among the upregulated DEGs common to all three comparisons were associated with the interferon response, immune cell activation, and the response to bacteria (Fig. 7A). Similarly, the upregulated DEGs in the OVX vs. E2 comparison were very highly enriched in immune-related pathways (Supplemental Fig. 7 A). Among the highest enriched pathways of the downregulated DEGs common to all comparisons, several were related to processing xenobiotics and metabolism related to different inflammatory lipids, such as arachidonic acid and eicosanoids (Fig. 7B). The downregulated genes in the OVX vs. E2 comparison were also related to pathways metabolizing lipids and xenobiotics, as well as transmembrane transport, likely linked to the substantial differential regulation of solute carrier genes in the E2-supplemented mice (Supplemental Fig. 7B). Overall, the enriched GO terms from the upregulated DEGs reflected the increased inflammatory response after E2-supplementation. GO analysis of DEGs found in the OVX vs. E2 + P4 comparison resembled the OVX vs. E2 analysis (Supplemental Fig. 7C-D), again indicating E2 as the strongest driver of inflammatory gene expression here.

**Fig. 8:**
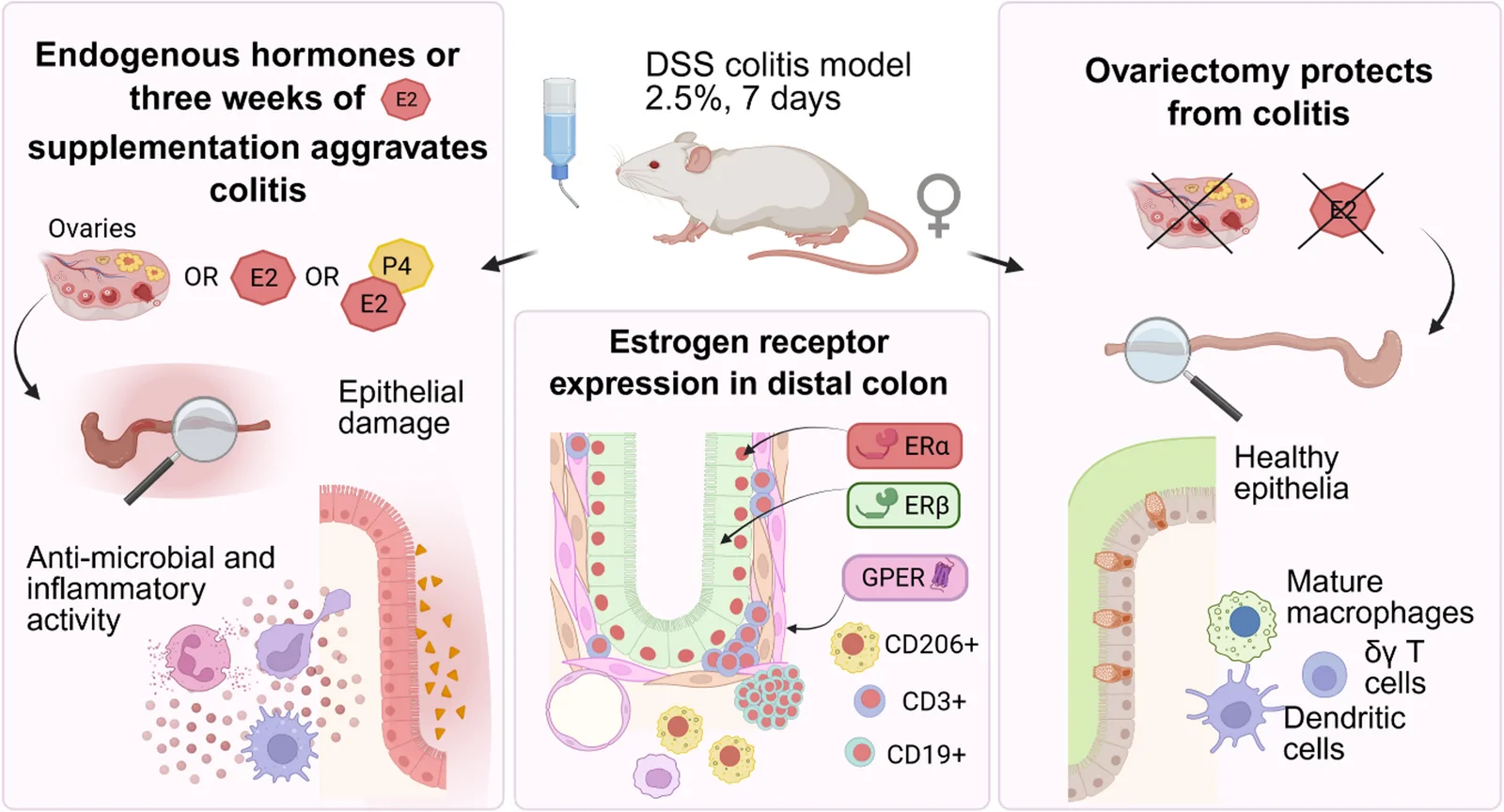
Estradiol or estradiol together with progesterone before induction of DSS-colitis aggravated inflammation in female mice. Endogenous hormones or three-week long pre-treatment with E2 or E2 + P4 after OVX increased epithelial damage in the distal colon after DSS challenge, as well as the antimicrobial response and neutrophil, monocyte and inflammatory macrophage immune response (left panel). OVX protected from inflammation and expanded dendritic cells, γδT cells and CX3CR1 + CD206+ macrophages (right panel). Estrogen receptor expression in the distal colon is depicted in the middle panel. ERα, represented by red nuclei in the different cell types, was expressed in colon epithelium, as well as in immune cells, while mucosal ERβ was observed only in the epithelium (represented by the green cytoplasm). GPER, represented by purple-colored cells, was observed in LP cells and in vessel walls, as well as in unidentified cells located in the submucosal edema

To summarize, in our results, E2 and E2 + P4, but not P4 alone, treatment for three weeks before induction of DSS colitis enhanced inflammation in female mice, and E2 exposure increased the epithelial damage and the innate immune response in colitis. Simultaneously, OVX protected from inflammation and expanded dendritic cells and known homeostatic immune populations, such as mature macrophages and γδT cells. Estrogen receptors were expressed in different cell types of the distal colon mucosa: ERα localized to nuclei in the colon epithelium, T and B lymphocytes, as well as in C206 + macrophages in submucosal edema. ERβ was expressed in the colon epithelium. GPER was found in unidentified cells located in submucosal edema, as well as in the LP and vessels (Fig. 8).

## Discussion

This study provided evidence of an aggravating effect of three-week E2 exposure before colitis. Also, we expand the understanding of the role of hormones in female colitis susceptibility and highlight the extensive response to E2 in the colon. Endogenous hormones or three-week E2 and E2 + P4 supplementation in OVX mice increased the colitis disease score, histological damage, expanded inflammatory immune cell populations, and increased expression of genes involved in epithelial and immune-related inflammatory pathways. These results were in line with earlier studies, where OVX was associated with decreased epithelial permeability and an eventual downregulation of cytokines compared to sham mice [43], and E2 pre-treatment of OVX mice potentiated the inflammatory effect of DSS [20, 21]. Even though P4 and progestins have been indicated to modulate inflammation [3, 35], pre-treatment with P4 alone did not influence colitis development in OVX mice and E2 + P4-treated mice did not differ significantly in inflammation from the E2 mice in any of these analyses. This was also in line with the only previous study regarding the role of progesterone receptor signalling before DSS colitis, where pre-treatment with progestogen alone or in combination with E2 did not influence the outcome [21]. It is possible that a longer experiment would have better recapitulated the increased IBD risk regarding contraceptive progestogens observed in women, but regarding E2, these results appear to agree with the results observed in human contraceptive use [3].

Even though E2- and E2 + P4-supplementation multiplied serum concentration of E2, compared to sham mice, the groups did not differ significantly in inflammation. In our analyses, the sham mice with physiological E2 fluctuation presented a similar effect compared to the E2-supplemented mice, although with less statistical power. The inflammatory E2 response might be saturated at a physiological high E2 concentration, thus a dose-response with significantly stronger inflammation did not occur in supplemented mice, although one could speculate that in a longer experiment, a higher prevalence of grave colitis would be observed in E2-supplemented mice, compared to sham. However, increased *Pgr* expression after E2 supplementation still indicated a stronger ERα transcriptional response after E2. A probable explanation is that all estrogen responsive cell types, such as the smooth muscle, do not participate in the inflammatory response. In addition to E2, E1 correlated with the inflammatory score. E1 has been associated with an inflammatory effect in obesity related chronic inflammation [61], thus it could play a role in the aggravated colitis seen here. However, sham mice presented increased inflammation without elevated E1, indicating that E1 did not add to the inflammatory effect. The increase likely originated from the high concentration of E2, which could drive the conversion of E2 to E1 [44]. Also, OVX induces weight gain and high-fat diet induced weight gain is often associated with more severe colitis [62], and mesenteric adipose tissue can signal in an inflammatory manner [63]. However, in this study, E2 increased inflammation independently of the metabolic effects of E2-depletion. It is possible, that the composition of the diet influences colitis risk to a greater extent than the increase of adipose tissue alone. On the other hand, also here, a longer experiment could have yielded a different outcome.

In previous results from the DSS mouse model by us and others, ERα signalling has been indicated to drive colitis [7, 20, 24], while ERβ has been suggested to be an important regulator of inflammation [9, 23]. ERβ has been reported as the most abundantly expressed estrogen receptor in the human colon [8]. Conversely, in our results, ERα was expressed at a higher level as transcripts, and it also had the broadest expression profile in the different cell types. Hence, future investigations into species differences in the relative ER subtype expression in the colon might be warranted. Nonetheless, the inflammatory effect of E2 signaling and ERβ receptor downregulation seen in mouse DSS colitis [20] seems to be relevant also to human disease, as it has been reported both in CD and UC [2, 3, 23, 64].

In these results, estrogen receptors were expressed in various cell types of the colon, including epithelial cells, LP, and immune cells, such as mature macrophages. Mature macrophages are selectively hyporesponsive to microbiota and, as such, serve as important mediators of tolerance by inducing an appropriate epithelial antimicrobial response [48, 49]. Inflammation induces a proportional shift in the differentiation stages of the monocyte-derived macrophage population in the colon, as the influx of inflammatory IL-1β-expressing monocytes is increased [54]. Here, the protective effect of OVX was reflected in the observation that the CX3CR1^hi^CD206 + mature macrophage population increased in E2-depleted mice, while E2 increased inflammatory activity. This, together with the coexpression of ERα and CD206, suggests that macrophages take part in the E2 inflammatory response. Previously, short in vitro studies have not recapitulated the inflammatory effects of E2 but have instead reported anti-inflammatory effects in macrophages [65, 66], which also was observed in a mouse model of asthma [67]. Nonetheless, after a 4-week E2 supplementation of OVX mice, extracted peritoneal macrophages were found to respond more strongly to LPS stimulation in an ERα-dependent manner [68]. Together with our results, this indicates that the chronic E2 response differs from the short exposure in macrophages, at least in the gastrointestinal system.

Outside the enteric nervous system, ERβ was observed in the cytoplasm of the distal colon epithelium. However, there are several considerations when investigating the distal colon localization of the low expression level estrogen receptors using antibodies. Even though we performed IHC staining and Western blot with positive and negative or low expression tissues, we cannot fully rule out unspecific staining of the antibody, which is a limitation to the study. In our results, ERα, but not ERβ, signal was nuclear in the colon epithelium. In contrast, previously the distribution of ERβ in the cytoplasmic and nuclear fractions was observed as equal in isolated colonocytes from healthy mice, while ERα signal was stronger in the cytoplasmic fraction [32]. In addition, even though ERβ was not observed in immune cells in these results, it is possible that the antibody signal did not reach detection threshold. Nonetheless, the intestinal epithelial-specific ERβ-depletion aggravated colitis [9], indicating that protective ERβ expression indeed is present in the colon epithelium.

GPER localized to vessel walls and to LP cells, possibly fibroblasts, which support epithelial regeneration by Wnt/β-catenin signalling and IL-33 [69, 70]. In the RNAseq dataset, several fibroblast-associated genes were upregulated, such as *Pfkfb3*,* Ccn5*,* Il33*,* Il11*, and *Saa3.* Previously, GPER activation has been reported to be anti-inflammatory in DSS studies with late hormone treatment [6]. The unspecified CD45^neg^CD11b^neg^F4/80^neg^ GPER+ cells located in submucosal edema was observed by both IMC and IF. As these cells did not express hematopoietic or myeloid lineage markers, they too might represent the mesenchymal lineage. Besides the plasma membrane, GPER can localize also to intracellular membranes, such as the endoplasmic reticulum, and perinuclear GPER expression has been reported in fibroblasts [10, 71]. In the IMC, perinuclear GPER was found also in F4/80 + CD206+ macrophages located in the submucosal edema, but IF results indicated only ERα in CD206 + macrophages. According to the literature, monocytes and macrophages express ERα, ERβ, and GPER [66, 68, 72]. However, unspecific staining cannot be ruled out here either as the control tissues were selected mainly according to ERα expression. While no ERα was observed in skeletal muscle, very low level ERβ and GPER were observed. In the literature, estrogen receptors have been suggested to be present in skeletal muscle, with some variation in the results, for instance, between males and females [15, 73, 74]. We used female mouse tissues for the GPER low expression controls, however, GPER expression in male skeletal muscle could have been even lower [15].

In addition to macrophages, intraepithelial γδ T cells are also crucial to intestinal homeostasis by selecting microbiota and supporting normal epithelial function. Disturbances in this short-chain fatty acid-regulated process are linked to severe Crohn’s disease [75], and γδ T cells can activate antigen-presenting CD11c+MHCII^hi^ DCs, which in turn can induce Th17 responses and potentiate inflammation [50]. γδ T cells are also known to be estrogen responsive in the uterus [76], but we are, to our knowledge, the first to report E2 depletion-associated γδ T cell expansion in the colon. However, our analysis was not sufficient to discover any potential differences in ER expression levels of specific cell types, or any differences in cell-type-specific receptor expression between treatment groups. ERα is expressed in T cells and B cells, both of which have been reported to respond to E2 [31, 77], and our mRNA dataset indicated downregulation of several B cell-associated genes. Detailed characterization of estrogen-responsive leukocyte subsets in acute and chronic colitis could offer an interesting research opportunity to explore their role in E2-aggravated chronic inflammation.

Also, an increase in CD11c+MHCII^hi^ DCs was observed in E2-depleted mice. The inflammatory potential differs between DC subsets, which induce a controlled inflammatory response, maintaining tolerance in the gut. Previously, specific ablation of the Clec9A+CD103 + DC subset was reported to worsen colitis. This was attributed to the lack of a controlled and reversible antimicrobial response by the intraepithelial lymphocytes, measured by upregulation of IFNγ response genes and *Ido1* in epithelia on day 4 of DSS [45]. Contrarily, here, the interferon response genes and IDO1 were highly expressed in E2-aggravated colitis on day 7 of DSS. Thus, differences between the onset of inflammation and active inflammation may explain the apparent mechanistic divergence and highlight the importance of an appropriate initial inflammatory response as a limiting factor in colitis.

Although ERα was observed in immune cells, the strongest nuclear ERα expression was detected in the colonic epithelium, consistent with recent reports of epithelial ERα activity in colitis [7, 32]. In intestinal homeostasis as well as in dysregulation, the constant communication of cells orchestrates the outcome, and epithelial-to-immune crosstalk is essential [78]. The intestinal epithelium secretes mucus and antimicrobial peptides, which form the outermost barrier against the microbiota [79]. The highest upregulated DEGs, *Clca4b* and *Ido1*, are both associated with colonocytes. *Clca4b* has been observed to be expressed in luminal colonocytes in response to inflammation, but its function remains unclear [80]. IDO1 function is well characterized in inflammation, and both deletion and pharmacological inhibition of IDO1 have been reported to attenuate DSS colitis [81]. Even though the literature suggests that E2 influences *Ido1* expression [82], no direct evidence has been presented for this in the colon, and the increase might be sequential to inflammation. Here, IDO1 was observed distinctly in the distal colon epithelium, which also expressed both ERα and ERβ. The strongest downregulated DEG, *Itln1*, is a microbial glycan-binding protein produced in goblet cells, and single-nucleotide polymorphisms in *Itln1* are associated with increased CD risk [83]. Here, ITLN1 was observed in intact luminal epithelium and in goblet cells. However, ITLN1 depletion in mice resulted only in a modest increase in susceptibility to DSS-induced colitis [84], and it is possible that the ITLN1 downregulation represents a less relevant transcriptional change, or that the decrease may be related to epithelial damage in colitis [85].

While interpreting the results, an evident challenge was to distinguish between inflammation-related and directly E2-induced alterations. The upregulated innate inflammatory cytokine IL-6 is a putative ERα target gene [59]; however, any E2-driven IL-6 expression is impossible to distinguish from the inflammatory cascade. Many of the other ERE-regulated genes found in our dataset play a role in different aspects of intestinal inflammation. For instance, the GO analysis of downregulated DEGs indicated pathways associated with the metabolism of xenobiotics, in which the enzyme CYP1A1 is involved. *Cyp1a1* polymorphisms resulting in loss of activity are associated with epithelial damage and have been reported to increase the risk of IBD [86]. The IDO1 system feeds into xenobiotic sensing and processing, which is activated by IDO1 inhibition [87]. Thus, IDO1 upregulation might decrease expression of genes related to this process, such as *Cyp1a1*, which are important for epithelial health and appropriate immune activation.

Other interesting DEGs with EREs were members of the proteolytic cascade, which activate coagulation, extracellular remodeling, cell motility, and the complement. These include upregulated genes *Plat*, *Plau*,* Plaur*, and *Serpine1*, as well as *F10* (coagulation factor X) and *C3*. tPA and uPAR were expressed in macrophages and neutrophils, respectively, located in the colitis-induced edema, which could be interpreted to affect cell motility and tissue remodeling during inflammation. In line with our results, *Plau* product urokinase plasminogen activator (uPA), which is active as a complex with its receptor uPAR, was previously observed in neutrophils [88]. Plasmin exacerbates cytokine release via matrix metalloproteinases, and previously, inhibition of uPA was reported to alleviate colitis in mice [88, 89]. Also, enzymatically inactive tPA has been suggested to inhibit excessive immune activation in colitis [90]. Here, tPA was observed in F4/80 + monocytes and macrophages. *Serpine1* and other serine protease inhibitors regulate the plasmin system by deactivating the plasminogen activators. The effects of this system are diverse, and inhibition of uPA and tPA inhibitor *Serpine1* or its product PAI-1 in mice has also alleviated DSS colitis by allowing tPA-mediated release of TGFβ [91], and *SERPINE1* expression was associated with active IBD [92].

To conclude, we show robust and repeated evidence [20] for an inflammatory effect of E2 pre-treatment, while short E2 treatment has alleviated inflammation in DSS colitis [6, 26–30]. We are the first to suggest the long E2 pre-treatment as a reason behind the reported divergent effects. E2-aggravated inflammation was associated with decreased epithelial repair response and increased barrier loss-associated antimicrobial activity, followed by inflammatory neutrophil and monocyte activity in the colon. Simultaneously, homeostatic cell populations, such as mature CX3CR1 + F4/80 + CD206+ macrophages, CD11c+MCHII^hi^ DCs, and γδ T cells were reduced. Based on our and others previous results, where ERα inhibition alleviated inflammation [20, 24], and the finding here that colon epithelial cells and different immune cell populations express ERα, we suggest that E2 exposure influences a broad range of processes to aggravate inflammation mainly via ERα activation, at least in female mice. The ERα-regulated epithelial damage response gene *Cyp1a1*, along with the plasmin system genes *Plat*,* Plau*,* Paur*,* Serpine1*,* F10*, and *C3*, may be noteworthy for future studies on E2 and ER signalling in inflammation. Additionally, the strongly downregulated ITLN1 relates to hormonally controlled processes.

This study underscores the importance of addressing the biological reasons behind the seemingly inconsistent results of E2 in different experimental colitis regimens. The divergence is possibly also observable in humans, but the phenomenon needs further investigation. In short, even though the contraceptive E2 increases IBD risk in pre-menopausal women [2, 3], hormone replacement therapy (HRT) using estrogen has been reported to alleviate symptoms of intestinal inflammation in post-menopausal women who were suffering from IBD before HRT [93]. Further investigations into the unintended effects of hormone use are warranted, especially regarding prolonged use of contraception or in treating PCOS, where ethinyl estradiol-containing contraceptives are used. The inflammatory risks may also extend to other estrogen receptor-active compounds, such as plastic softeners, which are common pollutants in the modern environment. BPA, a plastic softener compound with micromolar EC50 at both nuclear ERs [94], was reported to alter levels of microbial metabolites and aggravate DSS colitis in female mice [95]. Further, deepening our understanding of the kinetics of hormone-related inflammation and the diverse mechanisms behind the observed effects of E2 opens up novel research opportunities and could lead to new biomarkers or therapeutic options for inflammatory disease.

## Supplementary Information

Below is the link to the electronic supplementary material.

Supplementary Material 1 (PDF 789 KB)

Supplementary Material 2 (XLSX 15.9 KB)

Supplementary Material 3 (DOCX 8.43 MB)

## Data Availability

The RNAseq datasets supporting the conclusions of this article are available in the European Nucleotide Archive (ENA) at EMBL-EBI under accession number PRJEB96236. Other data is available from the authors upon request.

## Abbreviations

ANOVA: Analysis of variance
BW: Body weight
BSA: Bovine serum albumin
CD: Crohn's disease
CPM: Counts per million
DC: Dendritic cell
DSS: Dextran sulfate sodium
E1: Estrone
E2: Estradiol
ER: Estrogen receptor
FFPE: Formalin-fixed paraffin-embedded
GALT: Gut-associated lymphoid tissue
GAT: Gonadal adipose tissue
GO: Gene ontology
GPER: G protein-coupled estrogen receptor
HBSS: Hank´s buffered saline solution
Hi: High
HRT: Hormone replacement therapy
iFBS: Inactivated fetal bovine serum
IF: Immunofluorescence
IHC: Immunohistochemistry
IMC: Imaging mass cytometry
Int: Intermediate
LP: *Lamina propria*
OVX: Ovariectomy
P4: Progesterone
PCA: Principal component analysis
Perit: Peritoneum
PGR: Progesterone receptor
TNBS: 2,4,6-trinitrobenzenesulfonic acid
tPA: Tissue-type plasminogen activator
TPM: Transcripts per million
UC: Ulcerative colitis
uPA: Plasminogen activator urokinase
uPAR: Plasminogen activator urokinase receptor
