# Supplementary material for: Pre-Treatment with Estradiol, But not Progesterone, Exacerbates DSS Colitis: Dysregulated Innate Immunity and Impaired Epithelial Damage Response
Source: Inflammation. 2026 Jul 2;49(1):190. doi: 10.1007/s10753-026-02554-y (PMC13601184; doi:10.1007/s10753-026-02554-y)
Supplement: Supplementary file 1 [file 10753_2026_2554_MOESM1_ESM.pdf]

Supplemental Table 2. Differentially expressed genes per analysis.

DSS vs E2

Gene	Fold change
Clca4b	40,39
Ido1	38,09
Ltf	21,63
Reg3b	20,05
Reg3g	18,54
Iigp1	18,22
Lypd8l	16,08
Tgtp1	15,03
Saa3	13,84
Hp	12,97
Gbp2	12,64
Nos2	12,55
Gm12250	12,46
Cxcl9	12,27
Plet1	12,02
S100a9	11,06
Cxcl5	11,01
Igtp	10,89
Trim15	10,64
Gbp10	10,08
Tgtp2	9,98
Gbp6	9,70
Chil1	9,52
Gm4951	9,44
F830016B08Rik	9,39
Gbp4	9,33
Mmp10	8,94
Il11	8,83
Lrg1	8,82
Gbp5	8,74
Cxcl10	8,48
Ifi47	8,44
Cxcl2	8,29
Cemip	8,15
Mmp13	7,92
Mmp3	7,83
Lbp	7,81
Adamts4	7,75
S100a8	7,71
Ly6c1	7,46
Gm4841	7,45
Cxcl1	7,17
Irgm2	7,08
Il1b	7,04
Il18bp	7,02
Wfdc18	7,00
Sprr1a	6,87
BC023105	6,84
Gbp11	6,77
Socs1	6,61
Mfsd2a	6,53
Lcn2	6,43
Acod1	6,28
Duoxa2	6,28
Gbp3	6,21
Timp1	6,13
Serpina3f	6,03
Tnf	6,01
Gbp2b	5,93
Ly6a	5,88
Gbp8	5,84
Serpina3g	5,75
Socs3	5,67
Ccl8	5,58
Aldh1a3	5,55
Cxcl11	5,44
Ifit2	5,43

DSS vs E2P4

Gene	Fold change
Ido1	21,35
Reg3b	20,33
Clca4b	12,11
Saa3	11,32
Reg3g	10,80
Mmp10	9,55
Hp	8,97
Plet1	8,86
Ltf	8,76
Chil1	8,72
Il11	8,49
Sptssb	7,62
Mmp13	6,85
S100a9	6,69
Cxcl5	6,66
Cemip	6,29
Iigp1	6,29
Lrg1	6,02
Lypd8l	5,91
Adamts4	5,60
Trim15	5,50
Tgtp1	5,47
Nos2	5,39
Cxcl2	5,32
Il1b	5,14
Lcn2	5,11
Sprr1a	5,08
Mmp3	4,96
Gm12250	4,86
Wfdc18	4,68
Cxcl1	4,68
Bmp8b	4,63
Cxcl9	4,61
Timp1	4,56
Acod1	4,52
S100a8	4,46
Gbp2	4,38
Lbp	4,36
Il1rl1	4,29
Ly6c1	4,22
Igtp	4,20
Ereg	4,18
Gm4951	4,14
Tgtp2	4,12
Ccl8	4,08
Adgrf1	3,93
Gbp10	3,92
Ifit2	3,92
Gbp5	3,92
Wfdc12	3,88
Gbp6	3,84
Gbp4	3,82
Mfsd2a	3,79
Lgi2	3,78
Il18bp	3,74
Krt90	3,74
Pla2g2a	3,73
Cxcl10	3,72
Ly6a	3,70
Ifi47	3,69
Dio2	3,63
Tnf	3,63
Fgf23	3,63
Grem1	3,62
Duoxa2	3,62
Ccl7	3,60
Cxcl11	3,59

P4 vs E2P4

Gene	Fold change
Ido1	14,87
Clca4b	8,94
Reg3b	7,75
Sptssb	7,20
Plet1	7,03
Saa3	6,97
Il11	6,52
Hp	6,17
Ltf	6,06
Chil1	5,58
Nos2	5,51
Reg3g	5,26
Mmp10	5,25
Iigp1	5,04
Cxcl5	4,99
Adamts4	4,98
Tgtp1	4,94
S100a9	4,92
Cemip	4,58
Lrg1	4,54
Bmp8b	4,47
Lcn2	4,45
Mmp13	4,34
Gm12250	4,26
Trim15	4,24
Lypd8l	4,12
Mfsd2a	4,06
Sprr1a	4,05
Il1b	4,05
Cxcl2	4,01
Ereg	4,01
Igtp	4,00
Gm4951	3,90
Krt90	3,90
Ly6c1	3,84
Tgtp2	3,82
Mmp3	3,69
Gbp5	3,67
Ly6a	3,63
Ptx3	3,56
Cxcl1	3,56
Wfdc12	3,50
Ifi47	3,47
Dio2	3,46
Lbp	3,43
Gbp2	3,40
Ifit2	3,38
S100a8	3,38
Duoxa2	3,36
Cxcl10	3,30
Cxcl11	3,30
Wfdc18	3,30
Fgf23	3,29
Ccl8	3,28
Gbp4	3,25
Cxcl9	3,24
Acod1	3,22
Il1rl1	3,20
Gbp10	3,19
Socs3	3,15
Adgrf1	3,14
Myl7	3,12
Timp1	3,11
Pla2g2a	3,08
Alpl	3,05
Il1a	3,01
Chrdl2	3,01

Ptx3	5,41
Cd274	5,41
Ccl2	5,37
Lgi2	5,30
Zbp1	5,28
Nlrc5	5,27
Gm12185	5,18
NA	5,15
Fgf23	5,13
Alpl	4,99
Adgrf1	4,94
AW112010	4,93
Ereg	4,86
Hk3	4,86
Isg15	4,85
Dio2	4,85
Xdh	4,79
Oas3	4,74
Ccl7	4,70
Siglec1	4,68
Usp18	4,62
Tac1	4,62
Gbp9	4,51
Gbp7	4,48
Prss27	4,40
Il1rl1	4,40
Apol6	4,37
Tap1	4,34
Stat1	4,32
Grem1	4,25
Irgm1	4,18
Fcgr4	4,17
Pla2g2a	4,16
Ifi202b	4,14
Angptl4	4,07
Wars	4,07
Wfdc12	4,04
C3	4,02
Chrdl2	4,01
Csf3r	4,01
Oasl2	3,97
Psmb9	3,96
Fer1l4	3,96
Krt90	3,96
Nlrp3	3,95
Bmp8b	3,92
Bdkrb1	3,90
Psmb8	3,88
Tnfsf10	3,87
Serpina3n	3,86
Duox2	3,84
Igfbp5	3,83
Tnfaip2	3,81
Mt2	3,80
Lox	3,79
Lpl	3,78
Trim40	3,78
Sptssb	3,74
Calhm6	3,73
Adamts1	3,72
Nfil3	3,72
Ifi205	3,72
Fcgr1	3,70
Pla1a	3,69
Il6	3,68
Cxcr2	3,66
Ccl24	3,63
Selp	3,60
Lilr4b	3,59
Thbs1	3,58
Wfdc17	3,58

Ptx3	3,59
F830016B08Rik	3,57
Alpl	3,56
Socs3	3,46
Chrdl2	3,44
Il1a	3,43
Serpina3f	3,41
Prss27	3,38
Ccl2	3,37
Igfbp5	3,34
Mt2	3,31
Ccl3	3,28
Fer1l4	3,25
Gm4841	3,13
Myl7	3,12
Irgm2	3,11
Serpina3m	3,09
Angptl4	3,08
Gfap	3,04
Socs1	3,04
Isg15	3,02
Lpl	3,02
Gbp11	3,01
Siglec1	3,01
Il6	3,00
Wfdc17	3,00
Fcgr4	2,99
Gbp3	2,97
Clec4e	2,96
Tacr1	2,95
Marco	2,95
Serpina3n	2,94
AW112010	2,88
Serpina3g	2,88
Aldh1a3	2,86
Nlrp3	2,86
Ifi202b	2,85
Kcnj8	2,85
Ccl4	2,84
BC023105	2,84
Ptgs2	2,83
Ttyh1	2,83
Togaram2	2,82
Csf3r	2,81
Prss22	2,81
Gbp8	2,81
Gbp2b	2,81
Hk3	2,79
Zbp1	2,78
Tmem181c-ps	2,76
Xdh	2,75
Isx	2,72
Slc51b	2,72
Adamts1	2,72
Nfil3	2,72
C3	2,71
Il33	2,71
Epha2	2,70
Apol6	2,70
Bdkrb1	2,69
Bmper	2,67
Ifitm3	2,65
Duox2	2,63
Oas3	2,63
Usp18	2,63
Dhrs9	2,63
Ripk3	2,60
Krt36	2,60
Fst	2,59
Nlrc5	2,58
Ddah1	2,58

Serpina3m	3,01
Irgm2	2,97
Gbp6	2,95
Isg15	2,92
Grem1	2,90
Prss27	2,90
Fer1l4	2,87
Ccl7	2,86
Socs1	2,86
Gm4841	2,86
Isx	2,85
Il18bp	2,84
Serpina3f	2,81
Nfil3	2,79
Tnf	2,78
Igfbp5	2,77
Gbp8	2,70
F830016B08Rik	2,69
Adamts1	2,67
Gfap	2,66
Serpina3n	2,64
Slc51b	2,63
Ifi205	2,62
Lpl	2,62
Epha2	2,62
Ccl3	2,60
Spon1	2,59
Marco	2,58
Siglec1	2,58
Zbp1	2,56
Dhrs9	2,56
Tmem252	2,56
Gbp3	2,54
Clec4e	2,53
NA	2,52
Ptgs2	2,52
BC023105	2,51
Sphk1	2,50
Ccl2	2,50
Tacr1	2,49
Nt5c1a	2,49
Apol6	2,49
Slc4a11	2,48
C3	2,47
Prss22	2,47
Ttyh1	2,46
Smim3	2,46
Tmem181c-ps	2,45
Xdh	2,45
Trim40	2,45
Atg9b	2,44
Krt84	2,44
Il6	2,43
Duox2	2,43
AW112010	2,43
Togaram2	2,43
Hk3	2,42
Gbp2b	2,42
Lgi2	2,41
Ddah1	2,40
Gbp11	2,39
Oas3	2,37
Nrg1	2,37
Lif	2,35
Ccl4	2,35
Prrg4	2,35
Osmr	2,34
Nlrc5	2,34
Wfdc17	2,34
Ripk3	2,33
Gm12185	2,33

Upp1	3,56
Hdc	3,54
Ripk3	3,53
Il1a	3,52
Ifit1	3,51
Gm5431	3,51
Ptgs2	3,50
Clec4e	3,49
Dhrs9	3,47
Rsad2	3,46
Cfd	3,46
Kcnj8	3,45
Cwh43	3,43
Ifitm3	3,43
Clca4c-ps	3,42
Prg4	3,42
Osmr	3,40
Sfrp4	3,40
Serpina3m	3,40
Lif	3,40
Ces2f	3,36
Bmper	3,34
Rasl11a	3,34
Inhbb	3,30
Il22	3,29
Stom	3,27
Trem1	3,26
Prss22	3,26
Gfap	3,25
Car3	3,25
Tacr1	3,25
Il33	3,24
Batf2	3,23
Serpine1	3,21
Oas2	3,20
Ubd	3,20
Gm9640	3,18
Ccl4	3,17
Ccl11	3,16
Ccl3	3,16
Slc2a6	3,13
Spon1	3,13
Nt5c1a	3,12
Slc5a9	3,11
Duox1	3,11
Egr1	3,11
Trim30a	3,11
Sting1	3,10
Olfr56	3,10
Tgm2	3,08
Ifi44	3,06
Epha2	3,06
Ankrd1	3,05
Pfkfb3	3,04
Slc51b	3,03
Krt6b	3,03
Fst	3,02
Chl1	3,02
Tnfrsf1b	3,01
Mlkl	2,98
Ccn5	2,96
Slit3	2,95
Msr1	2,95
Pla2g5	2,94
Ifi27l2a	2,93
Apol10b	2,93
Ly6m	2,93
Tmem181c-ps	2,92
Marco	2,91
Ttyh1	2,90
Clec7a	2,90

Smim3	2,57
Trim40	2,55
Lif	2,55
Osmr	2,55
Ifi205	2,52
Dkk2	2,52
Ifit1	2,51
Hdc	2,50
Slc4a11	2,50
Oas2	2,48
Tac1	2,48
Sphk1	2,48
Spon1	2,48
Gm12185	2,47
Pla1a	2,47
Nt5c1a	2,47
Atg9b	2,45
Inhbb	2,44
Oasl2	2,44
Ier3	2,43
Apba2	2,43
Lpcat4	2,41
Cd274	2,41
Lilr4b	2,40
Thbs1	2,40
Creb3l3	2,39
Nrg1	2,39
Prrg4	2,38
Ctla2a	2,38
Wars	2,38
Psmb9	2,38
Cwh43	2,38
Bst2	2,37
Prg4	2,37
Ctps	2,36
Ccl11	2,35
Reg2	2,35
Krt84	2,35
Calhm6	2,34
Sulf2	2,33
Rsad2	2,33
Tnfaip2	2,33
Ms4a6d	2,33
Smpdl3b	2,33
Gvin-ps4	2,31
Stat1	2,30
Chl1	2,30
Vgf	2,30
Lama1	2,30
Tap1	2,29
Fcgr1	2,29
Clca4c-ps	2,29
Ecscr	2,28
Capg	2,27
Apol10a	2,26
Psmb8	2,26
Tgm2	2,26
Smoc1	2,26
C6	2,25
Ggt5	2,25
Lox	2,25
Irgm1	2,25
Slc5a9	2,25
Il4ra	2,24
Duox1	2,23
Gbp7	2,23
Serpine1	2,23
Samd5	2,22
Col18a1	2,22
Rasl11a	2,22
Stom	2,22

Lox	2,30
Oas2	2,30
Tnfsf10	2,28
Il33	2,27
Cwh43	2,27
Fcgr4	2,27
Kcnj8	2,26
Phf11d	2,26
Lpcat4	2,25
Ifitm3	2,25
Usp18	2,24
Irgm1	2,24
Apol7a	2,24
Bmper	2,24
Tac1	2,24
Ms4a6d	2,24
Stat1	2,22
Pla1a	2,22
Fosl1	2,20
Ctps	2,19
Serpina3g	2,19
Wars	2,19
Stom	2,19
Gbp7	2,17
C6	2,16
Smpdl3b	2,16
Prg4	2,16
Hdc	2,15
Capg	2,14
Tap1	2,14
Krt36	2,13
Oasl2	2,13
Gm5431	2,13
Bst2	2,12
Duox1	2,12
Mt2	2,12
Creb3l3	2,11
Angptl4	2,10
Rasl11a	2,10
Il4ra	2,10
Psmb9	2,10
Apol10b	2,09
Fst	2,09
Ifi44	2,08
Cd274	2,08
Gcg	2,08
Inhbb	2,08
Ifi202b	2,06
Tgm2	2,06
Apba2	2,06
Chl1	2,06
Ggt5	2,06
Hhipl1	2,05
Lilr4b	2,05
Cebpd	2,05
E2f2	2,05
Batf2	2,04
Pfkfb3	2,04
Bdkrb1	2,04
Ifitm1	2,03
Ngfr	2,03
Mmp9	2,03
Sulf2	2,03
Aldh1a3	2,03
Csf3r	2,02
Ctla2a	2,02
Tnfaip2	2,02
Cebpb	2,02
Msln	2,02
Fcgr1	2,02
Gem	2,02

C2	2,89
Bst2	2,89
Slc4a11	2,88
Cp	2,88
Smoc1	2,88
Ms4a6d	2,87
Ctps	2,87
Klra2	2,87
Krt84	2,87
Il4ra	2,86
Gvin-ps4	2,84
Krt36	2,84
Phf11d	2,82
Tap2	2,82
Clca3b	2,82
Nrg1	2,80
Myl7	2,78
Apol9a	2,78
Ifng	2,78
Gm20186	2,77
Pim1	2,76
Samd5	2,76
Snx10	2,75
Ly6g	2,75
Ddah1	2,75
Ecscr	2,75
Slpi	2,75
Apol7a	2,74
Zc3h12a	2,73
Samhd1	2,73
Cebpb	2,73
Smim3	2,72
Smpdl3b	2,72
Herc6	2,72
Slfn1	2,72
Clec10a	2,71
Ly6c2	2,71
Ier3	2,71
Ggt5	2,70
Phf11b	2,70
C6	2,69
Lilrb4a	2,67
Ngfr	2,67
Prdm1	2,67
Hcar2	2,66
Ifitm1	2,66
Igfbp4	2,66
Syt12	2,65
Prrg4	2,64
Togaram2	2,64
Plaat3	2,64
Ube2l6	2,63
Chrd	2,63
Capg	2,63
Gm14548	2,62
Hydin	2,62
Clec4d	2,62
Medag	2,62
Tifa	2,62
Eif4e3	2,61
Btnl2	2,60
Clca3a2	2,60
Irf1	2,59
Lama1	2,59
Ifit1bl1	2,59
Tnc	2,59
Plaur	2,58
Cxcl3	2,58
Adam12	2,58
Sulf2	2,58
Ifi204	2,58

Mmp9	2,21
Clip4	2,21
Slc3a1	2,19
Tspan4	2,18
Gem	2,18
Apol7a	2,18
Igfbp4	2,17
Mmp7	2,17
Trem1	2,17
Sema7a	2,16
Spon2	2,16
Fosl1	2,16
Phf11d	2,16
Btnl2	2,15
Gbp9	2,14
Pla2g5	2,14
Tnfsf10	2,14
Gpx2	2,14
Cxcr2	2,14
Cebpb	2,13
Egr1	2,13
Ces2f	2,13
Selp	2,13
Slit3	2,12
Plaur	2,12
Sting1	2,12
Upp1	2,12
Cck	2,12
Msr1	2,12
Cebpd	2,11
Inhba	2,11
Ifitm1	2,11
Snx10	2,11
Pfkfb3	2,10
Gcg	2,10
Tnc	2,09
Stc1	2,08
Birc5	2,08
Psat1	2,08
Ifng	2,08
S100a11	2,08
Tnfrsf1b	2,08
Mlkl	2,07
Chrd	2,06
Gpnmb	2,06
Cxcl16	2,06
Gpx2-ps1	2,06
Tgm1	2,05
Emp1	2,04
Sprr2a3	2,04
Slpi	2,04
Apol10b	2,03
Ifi44	2,03
Rrm2	2,03
Rnd1	2,03
Pim1	2,03
Gm5431	2,02
Pappa	2,02
Ccnb1ip1	2,01
Klra2	2,01
Hhipl1	2,01
Ccn5	2,01
Syt4	2,01
Clca3b	2,00
Slc34a2	-2,00
Methig1	-2,00
Ighm	-2,00
Htr4	-2,01
Hpn	-2,01
Fmo2	-2,01
Abcc6	-2,01

Btnl2	2,01
Spon2	2,01
Snx10	2,00
Col18a1	2,00
Gm5485	-2,00
Slc27a2	-2,00
Cd22	-2,00
Esr2	-2,00
Ngb	-2,01
Optc	-2,01
Bank1	-2,01
Slc37a2	-2,02
Retreg1	-2,02
H2-Eb2	-2,04
Rims4	-2,04
Ugt1a1	-2,06
Cyp2d12	-2,06
Tppp3	-2,07
Ighv3-8	-2,07
Gstm3	-2,08
Gm19980	-2,09
Per3	-2,09
Ces1e	-2,09
Cela1	-2,09
Hmcn1	-2,09
Hhip	-2,09
Ighg3	-2,09
Klb	-2,11
Cd37	-2,11
Iglc3	-2,12
Pdzd7	-2,12
Gpr174	-2,12
Gm15401	-2,12
Actc1	-2,13
Ugt2b35	-2,13
Cbs	-2,14
Reg4	-2,14
Blk	-2,14
Fut9	-2,15
Sycn	-2,15
Adh1	-2,17
Cyp2c69	-2,17
Siglecg	-2,19
Casp14	-2,19
Fcrl1	-2,20
Zan	-2,22
Ccr6	-2,24
Otc	-2,27
Cps1	-2,27
Cdh26	-2,28
Ttll6	-2,29
Ces1c	-2,29
Cxcr5	-2,29
Cyp1a1	-2,30
Tnfrsf13c	-2,30
Coq8a	-2,31
Mettl7a2	-2,31
Ighm	-2,32
Il22ra2	-2,33
Spib	-2,33
Dpyd	-2,35
Mettl7a3	-2,35
Kyat1	-2,35
H2-Ob	-2,36
Cyp2c40	-2,37
Chst3	-2,39
Sult1a1	-2,43
Anxa13	-2,46
Insrr	-2,50
Fgfr3	-2,51
Smpx	-2,51

Serping1	2,56
Apod	2,56
Icam1	2,56
Fpr2	2,55
Vgf	2,55
Clip4	2,55
Areg	2,54
Gfpt2	2,54
Cyp7b1	2,54
Apba2	2,54
Fosl1	2,53
Apol10a	2,52
Ly6i	2,52
Sphk1	2,52
Lpcat4	2,52
Tspan4	2,51
Spon2	2,51
S100a11	2,51
Col18a1	2,50
Cebpd	2,50
C4b	2,50
Cidec	2,49
Inhba	2,49
Ifi207	2,49
Trim10	2,49
Ptk6	2,48
Lap3	2,47
Sema7a	2,46
Hhipl1	2,45
Cxcl16	2,45
Gem	2,45
Stc1	2,44
Isx	2,44
C4a	2,44
Pgr	2,44
Ifit3	2,44
Sectm1a	2,43
Slc15a3	2,43
Tnip3	2,43
Pax8	2,43
C1rb	2,42
C1ra	2,41
Oasl1	2,41
Dmbt1	2,41
Sprr2a3	2,40
Mmp12	2,40
St3gal1	2,39
Npl	2,39
Ntrk2	2,39
Aldh1a2	2,39
Cfh	2,39
Itgb6	2,38
Sgk2	2,38
Tead4	2,38
Hgf	2,38
Itgam	2,38
Clca3a1	2,38
Krt13	2,37
Slfn5	2,37
Msln	2,37
Sod3	2,37
Gvin-ps2	2,37
Parp14	2,37
Gm21188	2,36
Tvp23a	2,36
NA	2,36
Lgals9	2,35
Anxa8	2,35
F10	2,34
Lamc2	2,34
Trafd1	2,33

Igkv3-2	-2,02
Igdcc4	-2,03
Actc1	-2,03
Ngb	-2,04
Klf12	-2,04
Ramp1	-2,05
Best2	-2,06
Per3	-2,06
Fcrl1	-2,07
Trpm6	-2,07
Upb1	-2,08
Pcp4	-2,08
Gm7902	-2,08
Cd79b	-2,09
Rims4	-2,09
Ctse	-2,11
Ms4a1	-2,11
Fmod	-2,11
Shisal1	-2,11
Timp4	-2,12
Aldh1a7	-2,12
Ikzf3	-2,13
Tgm3	-2,14
Cyp2c67	-2,14
Gm5485	-2,14
Adamts13	-2,15
Cyp2d12	-2,15
Pcdh17	-2,15
Klhdc7a	-2,16
Hexb	-2,16
Ces1f	-2,19
Ugt1a1	-2,19
Esrrg	-2,20
Gm15401	-2,20
Gstm1	-2,23
Adh1	-2,24
Hao2	-2,25
H2-Ob	-2,26
Coq8a	-2,26
Gstm3	-2,28
Kyat1	-2,29
Klb	-2,29
Tnfrsf13c	-2,30
Fcrla	-2,30
Tppp3	-2,30
Spib	-2,31
Hmcn1	-2,32
Cbs	-2,32
Il22ra2	-2,32
Gsta3	-2,33
Fut9	-2,33
Cps1	-2,33
Tent5c	-2,34
Casp14	-2,34
Fam222a	-2,37
Zan	-2,38
Cdh26	-2,38
Sycn	-2,39
Mettl7a3	-2,40
Reg4	-2,40
Odf3b	-2,41
Serpina9	-2,41
Hhip	-2,44
Pdzd7	-2,45
Slc37a2	-2,46
Lgr5	-2,47
Ugt2b35	-2,47
Cd19	-2,47
Cyp2c40	-2,50
Prr18	-2,50
Afm	-2,51

Afm	-2,53
Fcrla	-2,54
Cd79b	-2,61
Cyp2c68	-2,62
Ttr	-2,63
Ikzf3	-2,64
Slc30a10	-2,64
Tppp	-2,72
Ms4a1	-2,73
Itih2	-2,74
Lgr5	-2,78
A4gnt	-2,78
Cd79a	-2,91
Pck1	-3,04
Cr2	-3,17
Cd19	-3,18
Fcmr	-3,26
Cyp2f2	-3,31
Ighd	-3,47
Fcer2a	-3,78
Cyp2c55	-4,17
Glycam1	-4,57
Ang4	-4,59
Nxpe2	-4,90
Mptx2	-4,99
Mptx1	-5,30
Itln1	-8,43

Csf2rb	2,33
Plac8	2,33
Tnfrsf12a	2,32
Mgl2	2,32
Dtx3l	2,32
Fcer1g	2,32
Asap3	2,32
Plat	2,31
B430306N03Rik	2,31
Pdpn	2,31
Csf2rb2	2,31
Pira1	2,30
Mx1	2,30
Egr2	2,30
Arid5a	2,29
Sele	2,29
Tmem252	2,29
Rtp4	2,29
Ccr5	2,28
Alox15	2,28
Parp9	2,28
Gvin2	2,28
Cmklr1	2,27
Steap4	2,27
Clca4a	2,27
Stat2	2,27
Ifi211	2,27
Apol9b	2,27
Hmox1	2,26
Serpina3i	2,26
Cd14	2,26
Mmp9	2,25
Gch1	2,25
Hk2	2,25
Tgm1	2,25
Slamf8	2,25
C1s1	2,25
Il36g	2,24
S100a14	2,23
Dkk2	2,23
Adgrd1	2,23
Xaf1	2,22
Slfn4	2,22
Olfml2b	2,22
Ccl12	2,22
Vsig1	2,21
Pi15	2,21
Thpo	2,21
Pappa	2,21
Plau	2,21
Sh3kbp1	2,21
Sprr2a1	2,20
Ptges	2,20
Ch25h	2,20
Krt20	2,20
Adm	2,20
Gzmb	2,20
Rspo3	2,20
Plod2	2,19
Adcy4	2,19
Ccr1	2,19
Serpina10	2,19
Crem	2,18
Phlda1	2,18
Emp1	2,18
Fcgr3	2,18
Slc10a6	2,18
Sprr2a2	2,17
NA	2,17
Ctla2a	2,17
H2-DMa	2,17

Dpyd	-2,52
Fcmr	-2,54
Ttll6	-2,59
Cd79a	-2,60
Ces1c	-2,60
Angptl7	-2,61
Otc	-2,65
Anxa13	-2,67
Sult1a1	-2,69
Fgfr3	-2,71
Cr2	-2,78
Smpx	-2,78
Igkv3-4	-2,80
Slc30a10	-2,83
Cyp2c69	-2,85
A4gnt	-2,89
Ighd	-2,90
Ttr	-2,91
Ces1e	-2,96
Mettl7a2	-3,01
Cyp2c68	-3,02
Fcer2a	-3,07
Pck1	-3,08
Itih2	-3,31
Insrr	-3,57
Tppp	-3,66
Cyp2f2	-3,79
Glycam1	-4,26
Cyp2c55	-4,92
Nxpe2	-5,68
Ang4	-6,53
Mptx2	-7,46
Mptx1	-8,15
Itln1	-13,94

Gpx2-ps1	2,16
Psat1	2,16
Hbegf	2,16
Slc11a1	2,16
Plin1	2,15
Gpx2	2,15
Fas	2,15
Psmb10	2,15
Gvin1	2,14
Bcat1	2,14
Pde9a	2,14
Plekhn1	2,14
Psme2	2,14
Tbxas1	2,13
Gsap	2,13
Pirb	2,13
Aif1	2,13
Heg1	2,13
Art2a	2,13
Cd163	2,12
Tmem200a	2,12
Smox	2,12
Cers3	2,12
Gcg	2,12
Rnf213	2,12
Ly6g2	2,12
Gda	2,12
Rnf19b	2,12
Nckap5	2,12
Sp110	2,11
Rnd1	2,11
Igkv2-112	2,11
Lgmn	2,11
Myof	2,11
Sbno2	2,10
Ifit3b	2,10
Adora1	2,10
Dusp6	2,10
Lmcd1	2,10
Adamts5	2,10
Ccrl2	2,10
Tapbpl	2,10
E2f2	2,09
Efna5	2,09
Adipoq	2,09
Emilin2	2,09
Il1r2	2,09
Cd47	2,09
Tubb6	2,09
Cd300ld	2,09
Fam124a	2,09
Emb	2,08
Kirrel	2,08
Ackr2	2,08
Fgf10	2,08
Creb3l3	2,07
Ccl9	2,07
Tnfrsf1a	2,07
Dnah17	2,06
Epgn	2,06
Hspa2	2,05
Pde1a	2,05
B2m	2,05
Timd4	2,05
Gadd45g	2,05
Ctla4	2,05
Retnlg	2,05
Nxph3	2,05
C1s2	2,05
Pira2	2,04
Atg9b	2,04

Fut2	2,04
Lgals3bp	2,04
Sema6b	2,03
Cdc42ep2	2,03
Gpr146	2,03
Sprr2h	2,03
Rrm2	2,03
Fgf2	2,03
Ak7	2,03
NA	2,03
Clec4a3	2,03
F13a1	2,02
Slc6a2	2,02
Crispld2	2,02
Sh3pxd2b	2,01
Casp3	2,01
Vip	2,01
Rbp1	2,01
Gm8221	2,01
Plekho2	2,01
Abcc9	2,01
Ccl21b	2,01
Itga5	2,01
Gsdmd	2,00
Fbxw10	-2,00
Tspan33	-2,00
Npy1r	-2,01
Capsl	-2,01
Slc6a7	-2,02
Nr1i3	-2,02
Reep6	-2,02
Pbld2	-2,02
Aard	-2,03
Igkv8-21	-2,03
Sord	-2,03
Shank2	-2,04
Nxpe5	-2,04
Spag17	-2,04
Ces1b	-2,04
Pdk2	-2,04
Slc27a2	-2,05
Angptl7	-2,05
Zfp618	-2,05
Kcnh6	-2,05
Nr1h5	-2,05
Il22ra2	-2,06
Mettl7a1	-2,06
Osbpl6	-2,06
Tsku	-2,06
Fmo4	-2,06
Vtcn1	-2,06
Nqo1	-2,07
Kcne3	-2,07
Cldn2	-2,07
Hoga1	-2,07
Myo3a	-2,08
Slc2a13	-2,08
Lgals12	-2,08
Edn1	-2,08
Ppargc1a	-2,08
Stk26	-2,08
Hpd	-2,09
Cftr	-2,09
Lipt2	-2,09
Scin	-2,09
Hopx	-2,10
Mkx	-2,10
Hmga2	-2,10
Gpt	-2,11
Hid1	-2,12
Klhdc7a	-2,12

Cpm	-2,12
Gal3st2c	-2,12
Fcmr	-2,12
Cbr1	-2,13
Sfxn5	-2,13
Gm11735	-2,13
AI854703	-2,14
Fgf1	-2,14
Gm15998	-2,15
Lrrc31	-2,15
St6galnac2	-2,16
Slc16a12	-2,16
Slc35e3	-2,16
Gm20275	-2,16
Rnf186	-2,16
Fcamr	-2,17
Slc25a29	-2,17
Dio1	-2,17
Slc16a9	-2,18
Kif12	-2,19
Cacna2d2	-2,20
Ugt2b34	-2,20
Axin2	-2,20
Nxpe4	-2,20
Hoxa11os	-2,20
Gstm6	-2,21
Galm	-2,21
Ppara	-2,21
Fmo5	-2,22
Fmod	-2,22
Clca1	-2,22
Slc26a3	-2,23
B4galnt3	-2,23
Cela1	-2,23
Fcer2a	-2,23
St6galnac1	-2,23
Adamts13	-2,24
Sult1c2	-2,24
Klf12	-2,25
Stra6l	-2,26
St6gal1	-2,27
Arhgef19	-2,27
Kcnq1	-2,27
Cracr2a	-2,27
Ace2	-2,28
Tppp3	-2,28
Hdac11	-2,28
Akr1c19	-2,29
Ugdh	-2,29
Gvin-ps5	-2,30
Hykk	-2,31
Gstm7	-2,31
Pm20d1	-2,32
Sspo	-2,32
Aldob	-2,33
Ank	-2,33
Slc5a6	-2,33
Plekhd1	-2,34
Pdcd4	-2,34
Fam184b	-2,34
Dnah8	-2,35
Rbp2	-2,35
D630039A03Rik	-2,36
Cdh26	-2,37
Slc10a5	-2,37
Glycam1	-2,37
Aqp8	-2,38
Tinag	-2,38
Per3	-2,38
Acsl3	-2,38
Aldh1b1	-2,39

Ascl2	-2,39
NA	-2,40
Shisa2	-2,40
Cyp2s1	-2,40
Tmprss13	-2,42
Best2	-2,42
Tent5c	-2,42
Ybx2	-2,43
Smim24	-2,43
Mnx1	-2,43
Igdcc4	-2,46
Methig1	-2,46
Slc39a5	-2,47
Ldlrad3	-2,48
Selenbp1	-2,48
Mep1a	-2,48
Gvin-ps1	-2,48
Cyp2d13	-2,49
Esr2	-2,49
Selenbp2	-2,50
Cyp2w1	-2,51
Marcksl1-ps4	-2,51
Cyp2d26	-2,52
Aoc1	-2,52
Fndc5	-2,53
Cdo1	-2,53
Igkv3-4	-2,56
Gvin-ps3	-2,57
Abcb1a	-2,58
Ptpru	-2,59
Ramp1	-2,60
Aldh1a7	-2,60
Prr18	-2,60
Sytl1	-2,61
Tgm3	-2,61
Pcdh17	-2,61
Hexb	-2,61
Hunk	-2,62
Hpn	-2,62
Retreg1	-2,64
Rapgefl1	-2,66
Sycn	-2,68
Gstm1	-2,69
Acer1	-2,69
Abcc6	-2,72
Coq8a	-2,73
Mapk15	-2,74
Esrrg	-2,74
Edn2	-2,74
Rgs17	-2,79
Cyp2d12	-2,80
Upb1	-2,80
Timp4	-2,80
Trpm6	-2,80
Fam222a	-2,85
Ces1f	-2,87
Slc34a2	-2,87
Hmcn1	-2,87
Hhip	-2,88
Ugt1a1	-2,88
Rims4	-2,91
Slc20a1	-2,93
Fut9	-2,93
Cyp1a1	-2,94
Htr4	-3,02
Dpyd	-3,06
Ttll6	-3,06
Gm15401	-3,07
Mettl7a3	-3,09
Ces1c	-3,13
Odf3b	-3,14

Cda	-3,18
Rnf32	-3,18
Gm5485	-3,18
Tppp	-3,18
Casp14	-3,19
Zan	-3,28
Cyp2c67	-3,31
Clec2e	-3,33
Adh1	-3,37
Kyat1	-3,42
Serpina9	-3,44
Itih2	-3,45
Gstm3	-3,47
Sult1a1	-3,53
Anxa13	-3,60
Cps1	-3,64
Pdzd7	-3,64
Otc	-3,65
Klb	-3,70
Ugt2b35	-3,72
Cbs	-3,72
Hao2	-3,76
Slc37a2	-3,76
Fgfr3	-3,80
Lgr5	-3,86
Gsta3	-4,00
Smpx	-4,01
Afm	-4,08
Cyp2c40	-4,22
Mettl7a2	-4,25
A4gnt	-4,35
Reg4	-4,40
Ttr	-4,40
Insrr	-4,53
Pck1	-4,72
Ces1e	-4,84
Cyp2c69	-4,85
Cyp2c68	-5,65
Slc30a10	-5,83
Cyp2f2	-6,71
Cyp2c55	-11,36
Mptx2	-11,93
Ang4	-13,24
Mptx1	-13,41
Nxpe2	-13,77
Itln1	-20,52
