# Supplementary material for: Pre-Treatment with Estradiol, But not Progesterone, Exacerbates DSS Colitis: Dysregulated Innate Immunity and Impaired Epithelial Damage Response
Source: Inflammation. 2026 Jul 2;49(1):190. doi: 10.1007/s10753-026-02554-y (PMC13601184; doi:10.1007/s10753-026-02554-y)
Supplement: Supplementary file 2 [file 10753_2026_2554_MOESM3_ESM.docx]

# Supplementary information

**Supplemental Figure 1. Disease scoring, uterus weight, and serum hormone levels.** Uterus weight and appearance depict the E2 response, with lower weights and a decreased size in the OVX and OVX+P4 groups (A, B). Representative images of the uterus from sham (Bi), OVX (Bii), E2 (Biii), P4 (Biv), and E2+P4 (Bv). E1 was increased in the serum of E2-supplemented mice (C), and serum P4 was decreased in the OVX and E2 groups (D). The BW increases during two weeks before starting DSS treatment was significantly greater in the OVX mice, as well as the P4 and E2+P4 treated mice when compared to sham (E). Fecal (stool consistency, F) and intestinal bleeding (G) scores decreased in E2-depleted mice. These were combined with BW change into an endpoint disease score (H, experiment 1, and I, experiment 2). Serum E1 concentration weakly correlated with the total inflammation score (J), while serum P4 did not correlate with inflammation (K). Sham n=8, OVX, E2, P4, E2+P4 n=14 in each group in A-F, G n=38, H n=38. Data presented as mean and SD. Statistical analysis using one-way ANOVA (A, C-E, H-I) or two-way ANOVA (F-G), together with Tukey´s post-hoc test.

**Supplemental Figure 2. Flow cytometry information.** The gating strategy of adaptive and innate immune cells. Single cells were distinguished from debris by size before selecting the live cell population and gating the cells by immune marker expression (A). Representative image CD45+CD11b+Ly6G^neg^MHCII+F480+CX3CR1hiCD163+ macrophages, which were not altered in the E2-aggravated inflammation (B).

**Supplemental Figure 3.** Relative expression of Esr1 (A), Esr2 (B), Gper1 (C), and Pgr (D) from experiment 2. Cq and TPM values of the hormone receptor genes showing Esr1 mRNA as the most abundant, and Esr2 or Gper1 as the least abundantly expressed estrogen receptor in the distal colon. Pgr baseline expression was even lower, and increased in the E2-supplemented group (RT-qPCR from exp 1(black bars) and 2 (white bars) in E, RNAseq from exp1 in F). Sham n=5, OVX n=4, E2, P4, E2+P4 n=6/group). Data is presented as mean and standard deviation. Statistical analysis was performed with one-way ANOVA and tested for multiple comparisons. Representative images of ERα blotted distal colon protein from three separate membranes (G).

**Supplemental Figure 4.** ERα (Ai and ii) and ERβ (Bi and ii) antibody signal in individual tissue samples from two healthy female mice. GAPDH was used as loading control. The order of the sample corresponds to that indicated in the antibody signal quantification of ERα (C) and ERβ (D) in mouse tissue Western blots, showing the expected tissue distribution of ERs with stronger signal in reproductive tissues (ERα) and adipose tissue (ERβ) (GAT = gonadal adipose tissue, perit = peritoneum). Representative images of FFPE control tissue IHC staining of mouse vaginal (overview, blue circle) epithelium and stroma, showing clear ERα (i) and ERβ (ii) positive cells, while GPER (iii) signal is present but weaker. Secondary antibody control IHC staining of mouse vagina anti-rabbit IgG and anti-mouse IgG antibodies (iv and v, E). Representative images of skeletal muscle control staining of ERα, showing no signal, ERβ, showing nuclear signal and GPER, showing weak cytoplasmic signal (F, overview and i-iii, respective IF staining). Representative images of negative control IF staining in distal colon for ERβ with anti-mouse IgG Alexa488 secondary antibody only, showing mouse IgG unspecific signal in green. This unspecific signal was excluded from the ERβ results (G i, ii). Rabbit IgG 1:100 as a control for ERα and GPER on frozen section together with anti-rabbit secondary antibody conjugated to Alexa647 in crypts (H i-ii) and GALT (H iii). Negative control staining for rat anti-mouse antibodies (CD3, CD19), with anti-rat IgG secondary antibody conjugated to Alexa586 in GALT and crypts (I i, ii). Rabbit IgG 1:200 on paraffin section, together with anti-rabbit secondary antibody conjugated to HRP (J). Orange arrowhead indicates intestinal crypts, and yellow arrowhead indicates GALT.

**Supplemental Figure 5. Explorative imaging mass cytometry of the inflamed colon.** Representative HE staining of the region of interest (A). GPER+ cells in submucosal edema were either CD45^neg^CD11b^neg^ (orange arrows) or CD45^low^CD11b+ (cyan arrows) (B). Submucosal edema located GPER+CD45^neg^CD11b^neg^ cells (orange arrows) and GPER+CD45^low^CD11b+ cells, which were also F4/80+ but CD80^neg^ (cyan arrows) (D, and higher magnification in E). GPER+F4/80+ cells were also CD206+ (E). n=1, 3 regions of interest. Pseudocolors GPER-magenta, CD45-red, CD11b-yellow, nucleus-blue, CD45-red (B), F4/80-red (C-E), CD80-green (D), and CD206-green (E).

**Supplemental Figure 6.** IDO1 was expressed in the epithelium of OVX (A) and E2-supplemented mice (B), with increased protein expression in E2 mice (C-D). Similarly, COX2 was increased in E2-supplemented mice (E-F). ITLN1 was increased in OVX mice (G-H). Guiding image of the areas depicted in J-M, the yellow box indicates epithelium, and the black box indicates submucosal edema (I). ITLN1 was expressed in intact luminal epithelium as well as goblet cells (pink arrowheads, J), while this was lost in damaged epithelium (K). tPA was observed in the submucosal edema F4/80+ cells (green arrowheads), but not as strongly in neutrophils (orange arrowheads, L). uPAR expression was strong in neutrophils located in the submucosal edema (orange arrowheads), while the F4/80+ cells did not express uPAR to a similar extent. All sections from experiment 1. Data is presented as mean and standard deviation. Statistical analysis was performed using one-way ANOVA with Tukey´s test for multiple comparisons (C, F, H). A-F: sham n=2, OVX, E2, P4, E2+P4 n=8/group. G-H: OVX, E2, P4, E2+P4, n=3/group. J-M: OVX and E2, n=3/group.

**Supplemental Figure 7.** GO of the upregulated (A) and downregulated (B) DEGs in the OVX vs E2 comparison, and of the upregulated (C) and downregulated (D) DEGs in the OVX vs E2+P4 comparison. OVX, E2, P4, E2+P4 n=8 in each group.
